# Oral streptococci: modulators of health and disease

**DOI:** 10.3389/fcimb.2024.1357631

**Published:** 2024-02-22

**Authors:** Susanne Bloch, Fiona F. Hager-Mair, Oleh Andrukhov, Christina Schäffer

**Affiliations:** ^1^ Competence Center for Periodontal Research, University Clinic of Dentistry, Medical University of Vienna, Vienna, Austria; ^2^ Department of Chemistry, Institute of Biochemistry, NanoGlycobiology Research Group, Universität für Bodenkultur Wien, Vienna, Austria

**Keywords:** biofilm, host interaction, oral diseases, *Streptococcus* sp., virulence factors

## Abstract

Streptococci are primary colonizers of the oral cavity where they are ubiquitously present and an integral part of the commensal oral biofilm microflora. The role oral streptococci play in the interaction with the host is ambivalent. On the one hand, they function as gatekeepers of homeostasis and are a prerequisite for the maintenance of oral health - they shape the oral microbiota, modulate the immune system to enable bacterial survival, and antagonize pathogenic species. On the other hand, also recognized pathogens, such as oral *Streptococcus mutans* and *Streptococcus sobrinus*, which trigger the onset of dental caries belong to the genus *Streptococcus*. In the context of periodontitis, oral streptococci as excellent initial biofilm formers have an accessory function, enabling late biofilm colonizers to inhabit gingival pockets and cause disease. The pathogenic potential of oral streptococci fully unfolds when their dissemination into the bloodstream occurs; streptococcal infection can cause extra-oral diseases, such as infective endocarditis and hemorrhagic stroke. In this review, the taxonomic diversity of oral streptococci, their role and prevalence in the oral cavity and their contribution to oral health and disease will be discussed, focusing on the virulence factors these species employ for interactions at the host interface.

## 1 Introduction

Streptococcal species accompany us throughout our live – in the oral cavity, they are the first colonizers after birth and, onwards, they shape the establishment of a complex microbiota in health and disease (Abranches et al., 2018; Klein, 2022). Their interactions with the human host range from highly beneficial – influencing, as commensal species, proper immune system development (Lang et al., 2010) and preventing pathogen colonization - to detrimental, when dissemination in the blood stream occurs, potentially leading to no less than infective endocarditis (IE), purulent infections, brain hemorrhage, intestinal inflammation, autoimmune diseases, and bacteremia, among others (Yumoto et al., 2019). This review focuses on oral streptococci, their taxonomic diversity and roles within their ecological niche, and, first and foremost, their interaction with the host, within the oral habitat and beyond. Complementary to this review, methods and tools for the detection, identification, characterization, and spatial localization of oral streptococci have been summarized in a recent book chapter (Klein, 2022).

## 2 Taxonomic overview of oral streptococci

Streptococci are facultative anaerobic, Gram-positive bacteria. Historically, species within the genus *Streptococcus* were classified according to their hemolytic potential on blood agar plates as fully (β-)hemolytic, partially (α-) hemolytic and non- (γ-) hemolytic (Sherman, 1937). β-hemolytic streptococci were further subcategorized according to the carbohydrate composition of the antigens present in their cell wall (Lancefield groups) as Group A *Streptococcus* (GAS), such as the highly pathogenic *Streptococcus pyogenes*, or as Group B *Streptococcus* (GBS), such as *Streptococcus agalactiae* (Facklam, 2002; Abranches et al., 2018).

With the advent of *16S rRNA* sequencing techniques, the genus *Streptococcus* was further categorized into eight distinct groups based on phylogenetic relationships between its members; these are the *mitis*, *sanguinis*, *anginosus*, *salivarius*, *downei*, *mutans*, *pyogenic*, and *bovis* groups (Abranches et al., 2018). While the *mitis* group constitutes the largest group with 20 members detected in the oral cavity, oral streptococci – also often referred to as *viridans* streptococci – cluster in all groups except for *bovis* and *pyogenic* (Abranches et al., 2018). Between these groups as well as species within the same group, differences in virulence and pathogenicity can be observed (Sitkiewicz, 2018). For instance, members of the *mutans* group, *e.g.*, *Streptococcus mutans* and *Streptococcus sobrinus*, have been identified as species with high cariogenic potential (Richards et al., 2017). *Mitis* group streptococci (MGS) such as *Streptococcus mitis*, *Streptococcus oralis*, *Streptococcus gordonii*, *Streptococcus infantis, Streptococcus sanguinis*, and *Streptococcus parasanguinis*, on the other hand, are associated with oral health (Richards et al., 2017). Notably, especially the first three species have recently been identified as causative agents in the development of IE, after the occurrence of a blood stream infection (Chamat-Hedemand et al., 2020). Among others, *S. mitis* as well as *S. oralis* have recently been classified as members of the core oral microbiota, *i.e.*, they belong to a group of oral bacteria that is ubiquitously present within the oral cavity (Hall et al., 2017). When it comes to their pathogenic potential, MGS as well as *Streptococcus anginosus* Group (SAG) members have long been considered as commensals integrated in a healthy human microbiota, however, they have more and more frequently been recognized to elicit significant health problems in humans (Mitchell, 2011; Kuryłek et al., 2022; Pilarczyk-Zurek et al., 2022).

## 3 The oral cavity: habitat for complex polymicrobial communities in health and disease

The oral cavity is a unique and multifaceted bacterial habitat with distinct ecological niches, resulting from the presence of different surfaces for colonization and varying conditions due to considerable fluctuations in oral environmental parameters such as temperature, pH, redox potential and nutrient availability, which, in combination with behavioral aspects of the human host (*e.g.*, dental hygiene, diet, smoking) as well as genetic predisposition and the general health status, shape the composition of the resident microbial consortia (Mark Welch et al., 2020). The non-shedding surfaces of the teeth, the dorsal and lateral surfaces of the tongue, the periodontal pockets, and the remaining epithelial surfaces of the oral mucosa offer sites for colonization by distinct microbes. Streptococci have been found to be present at all of these sites and to be the dominant genus in supra- and subgingival plaque and on soft tissues (Mager et al., 2003; Aas et al., 2005; Huse et al., 2012; Mira, 2018). As primary/pioneer colonizers, they are the first species to colonize the oral surfaces and allow for the establishment of other species such as *Actinomyces*, *Veillonella*, *Fusobacterium*, *Prevotella*, and *Neisseria*, initializing the process of microbial succession and establishment of a complex microbial consortium (Sampaio-Maia and Monteiro-Silva, 2014; Gomez and Nelson, 2017). Recent investigations revealed a new understanding of how the oral biofilm is formed from bacterial aggregates serving as nuclei for the development of a strong biofilm embedded in the extracellular matrix. Regarding the presence of streptococci, an interesting observation was made concerning their localization; in addition to early colonization at the biofilm basis, these were detected radially in the outermost layer of the biofilm providing a microenvironment for strict anaerobes by consuming present oxygen (Simon-Soro et al., 2022).

Formation of dental plaque on the non-shedding surfaces of the teeth is one of the best characterized multispecies biofilm community activities; dental plaque is present in healthy individuals, but it is also associated with the development of oral diseases, including caries, gingivitis and periodontal diseases (Marsh and Zaura, 2017).

### 3.1 Dental caries

Streptococci are the predominant species to initially adhere to the salivary pellicle (Diaz et al., 2006). In supragingival plaque, predominantly the dietary intake of sugars promotes the formation of extracellular polymeric substances (EPS) and acidic metabolites causing the resident microflora to shift towards aciduric and acidogenic species (Lamont et al., 2018). If persistent, this leads to the acidification of the biofilm microenvironment and, ultimately, to the demineralization of dental enamel and the development of carious lesions (Lamont et al., 2018). The role of *mutans* streptococci (MS), *i.e.*, *S. mutans* and *S. sobrinus*, as cariogenic agents has long been recognized (Conrads et al., 2014; Hajishengallis et al., 2017). The cariogenic potential of MS stems from three characteristic traits – the species’ ability to synthesize large quantities of extracellular glucan from sucrose, their ability to metabolize a wide range of carbohydrates into organic acids, *i.e.*, their acidogenicity, and their tolerance towards environmental stress conditions such as low pH, *i.e.*, their aciduric nature (Lemos et al., 2019). Extracellular glucans form essential building blocks of the EPS as part of the extracellular biofilm matrix, which confers to the bacteria protection from shear forces and resistance to antimicrobials, influences the diffusion of oxygen, nutrients, quorum sensing signals and metabolites, and creates an acidic microenvironment in which commensal bacteria are outcompeted by cariogenic species (Bowen et al., 2018).

### 3.2 Pulpitis and endodontic infections

Carious lesions or other factors that damage the tooth’s integrity can reach the dental pulp, which is principally encased in the root canal system, exposed to the bacteria present in the oral cavity. Bacterial colonization elicits inflammation and necrosis of the dental pulp as well as inflammation of the periapical region, finally leading to bone resorption and the formation of granulomas or cysts (Graves et al., 2011). Oral streptococci, such as *S. oralis*, *S. anginosus*, *S. mitis*, *S. sanguinis* (Chávez de Paz et al., 2005; Narayanan and Vaishnavi, 2010), and *S. mutans* (Lima et al., 2020) are frequently among the species detected in root canal infections. In the dental pulp, bacterial pathogen associated molecular patterns (PAMPs) are recognized by macrophages, dendritic cells (DCs), odontoblasts and endothelial cells, and elicit an immune response leading to the recruitment of specialized immune cells and the initiation of bacterial clearance (Khorasani et al., 2020). The inflammatory response mechanisms, cytokine networks as well as pulpal disease pathogenesis are the focus of other reviews (Hahn and Liewehr, 2007a; Farges et al., 2015; Khorasani et al., 2020; Galler et al., 2021).

### 3.3 Periodontal diseases

The onset of gingivitis and emergence of periodontal diseases is caused by the shift in the composition of the resident oral microbiota from commensal to dysbiotic (Hajishengallis and Lamont, 2012). In a healthy individual, bacteria in the subgingival crevice exist in a balanced state with the host immune system, under controlled, low-level inflammation keeping bacterial growth in check (Hajishengallis and Lamont, 2016). This symbiotic state can be disrupted if certain risk factors, such as disadvantageous host genetics or lifestyle, or systemic diseases apply, or if keystone pathogens are present (Loos and Van Dyke, 2020). Dysbiosis is characterized by an increase in bacterial mass and the prevalence of Gram-negative, anaerobic species, leading to chronic inflammation (Hajishengallis et al., 2020). Particularly, the emergence of inflammophilic species such as *Porphyromonas gingivalis*, *Treponema denticola*, *Tannerella forsythia*, *Aggregatibacter actinomycetemcomitans* or *Filifactor alocis* (Haffajee et al., 2008; Könönen et al., 2019), their modulation of the immune response and interaction with the resident polymicrobial community support an exacerbation of inflammation and bacterial overgrowth (Hajishengallis et al., 2012). Periodontal pathogens are capable of evading (Hajishengallis, 2011; Bloch et al., 2018, 2019) and subverting (Hajishengallis and Lamont, 2014; Hajishengallis and Diaz, 2020) the host immune response and thereby dysregulate immune homeostasis. A detailed description of immune mechanisms in the context of periodontal diseases can be found in several excellent reviews (Hajishengallis, 2014; Ebersole et al., 2017; Könönen et al., 2019).

### 3.4 Oral streptococci and their balancing act between health and disease

Oral streptococci regulate the structure and function of the oral microbiome in a way that is beneficial to human health (Baty et al., 2022). However, *Streptococcus* was determined by metagenomic analyses as the predominant genus in patients with gingivitis (Park et al., 2015). As pioneer colonizers, streptococci not only enable later colonizers and pathogenic species to co-adhere, they also can be considered as accessory pathogens, as is the case for *S. gordonii* whose interaction with *P. gingivalis* and *A. actinomycetemcomitans* was found to substantially elevate these species’ pathogenicity (Kuboniwa et al., 2006; Daep et al., 2011; Ramsey et al., 2011). In pathogen-free mice, inoculation with *P. gingivalis* led to a massive increase in streptococcal cell numbers (Hajishengallis et al., 2011) and in an *in vitro* subgingival biofilm model, omission of the periodontal pathogen from the community resulted in invasion of gingival epithelial cells by *S. oralis* instead (Thurnheer et al., 2014). On the other hand, *P. gingivalis* failed to cause periodontal disease in germ-free mice in the absence of the commensal core microbiome –which in fact was capable of causing modest bone loss in germ-free mice on its own putting oral streptococci in the spotlight as modulators of oral immune responses (Hajishengallis et al., 2011; Abusleme et al., 2013). These findings highlight the central role oral streptococci play in the balancing act between the maintenance of host-microbiome homeostasis during health and immunomodulatory effects in disease. In the following sections, the factors employed by these bacteria to tip this balance towards a healthy or diseased state will be discussed.

## 4 Immunomodulatory effects exerted by commensal oral streptococci

**Figure 1:**
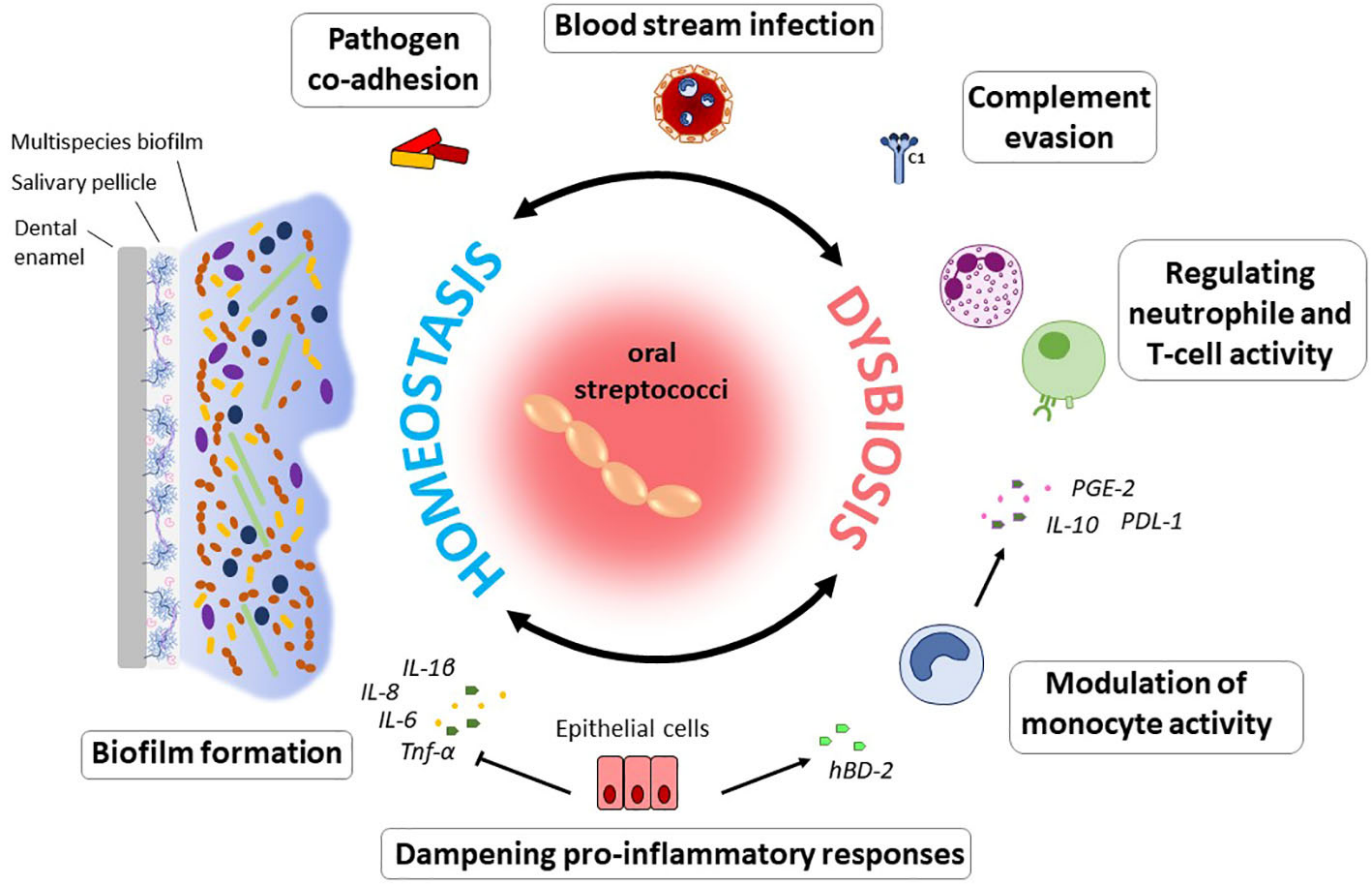
Immunomodulatory effects of oral commensal streptococci. The ability to form biofilms on numerous substrates can be regarded as a hallmark of oral streptococci. From within oral biofilms, streptococci interact with immune and epithelial cells, enable pathogens to co-adhere, and can disseminate into the blood stream. Modulation of the immune response to the bacterial biofilm challenge enables their persistence and survival in the host and determines the balance between immune homeostasis and dysbiosis. Specifically, through the downregulation of pro-inflammatory immune responses, oral streptococci contribute to a balanced immune status. Induction of hBD-2 released *e.g.*, by *S. mitis* counteracts colonization by pathogenic species. This commensal was also found to induce a pro-inflammatory immune response in monocytes, while at the same time inhibiting neutrophil activity and T cell proliferation through the action of IL-10, PD-L1 and PGE2. In the context of systemic disease, it is beneficial for many streptococcal species that they can evade complement mediated immunity and thereby remain under the radar. Especially in periodontal disease, the role of oral streptococci to adhere to numerous substrates as primary colonizers and biofilm builders makes them a contributing factor to disease development, since they enable colonization of pathogenic species.

Maintenance of homeostatic control of infection and inflammation is crucial to oral health. As dominant members of the resident oral microflora, streptococci exert essential functions in this process (**Figure 1**). In a study by Myers and coworkers, 30% of streptococci isolated from supra- and subgingival plaque samples were found to exhibit anti-inflammatory properties, specifically by downregulation of IL-8 production in epithelial cells (Myers et al., 2021). Operating as an oral commensal, *S. gordonii* can supress the secretion of IL-6 and IL-8 in epithelial cells, which contrasts with *Fusobacterium nucleatum*, an important bridging bacterium in oral biofilms that is regarded as a potential pathogen and was examined in the same study (Hasegawa et al., 2007). Other species such as *S. salivarius* or *S. mitis* contribute to a balanced immune status by attenuating pro-inflammatory immune responses towards themselves as well as towards pathogenic species (Cosseau et al., 2008; Eberhard et al., 2009; Sliepen et al., 2009a). Furthermore, *S. mitis* induces the release of the antimicrobial cationic peptide human β-defensin 2 (hBD-2) which is not harmful to *S. mitis* itself, but affects other bacteria (Eberhard et al., 2009; Mitchell, 2011) and thereby antagonizes oral colonization by pathogenic species. Expression of hBD-2 has been found to be upregulated in gingival epithelial cells in response to commensals, *e.g.*, *F. nucleatum*, but not to pathogenic species, such as *P. gingivalis*, and is produced in the gingiva also in a non-inflamed state (Krisanaprakornkit et al., 2000). Apart from bacterial killing, hBD-2 can also inhibit bacterial biofilm formation at nanomolar concentrations, as was shown for, *e.g.*, *Pseudomonas aeruginosa* and *Acinetobacter baumannii* (Parducho et al., 2020). In response to *S. mitis* challenge, monocytes produce chemotactic as well as pro-inflammatory mediators and at the same time increase the secretion of interleukin-10 (IL-10) and prostaglandin E2 (PGE2) and expression of programmed cell death protein ligand 1 (PD-L1), inhibiting neutrophil activity and T cell proliferation (Engen et al., 2018). *S. mitis* can, thus, trigger the recruitment of immune cells to the site of infection, while at the same time dampening the inflammatory response and thereby promote commensal bacterial tolerance and survival on site. Certain MGS and *sanguinis* streptococci, especially those associated with systemic infections, even display immune evasion mechanisms being less susceptible to complement opsonization and thereby escape immune surveillance (Alves et al., 2019). In *S. mutans*, complement resistance as well as uptake by neutrophils is controlled by the orphan response regulator CovR, a repressor of virulence factor expression (Negrini et al., 2012; Alves et al., 2016). Specifically, CovR regulates the expression of genes involved in cell wall biogenesis and surface interactions with EPS and impacts not only susceptibility to complement opsonization and survival in blood, but also the formation of cariogenic biofilms on the tooth surface (Alves et al., 2016).

## 5 Streptococcal adhesion and initial colonization

The salivary pellicle that forms on the tooth and mucosal surfaces constitutes the primary substrate for streptococcal colonization *via* specialized adhesins, which can bind to albumin, proline-rich proteins, glycoproteins, mucins, and sialic acid (Sia) (Abranches et al., 2018). Other structures that oral streptococci are able to adhere to include glucan, collagen, plasminogen, laminin, and fibrinogen (Nobbs et al., 2009). Streptococcal adhesion and colonization has been reviewed in detail by Nobbs et al. (Nobbs et al., 2009) and will be briefly discussed here.

Anchoring of streptococcal cell surface proteins typically occurs *via* their Leu‐Pro‐x‐Thr‐Gly (LPxTG) motif and is facilitated by sortases that catalyze the attachment of the proteins to the cell wall (Okahashi et al., 2022). The cell-wall anchored antigens I/II (AgI/II) – also named P1, SpaB, AgB, or PAc - belong to a family of adhesins that is present in most oral streptococci, and recognizes multiple host proteins thereby facilitating sucrose-independent biofilm formation, platelet aggregation, invasion of tissues, and interaction with the host immune system (Koga et al., 1990; Brady et al., 2010; Abranches et al., 2018). AgI/II proteins have been found to bind to fibronectin, salivary glycoproteins and proline-rich proteins, collagen, laminin, fibrinogen, platelets and α_5_ß_1_ integrin on epithelial and endothelial cells (Nobbs et al., 2009). *S. gordonii* AgI/II proteins SpaA and SpaB have also been shown to facilitate interaction with oral *Actinomyces* species (Jenkinson et al., 1993) and *P. gingivalis*, enabling invasion of dentinal tubules by the periodontopathogen (Love et al., 1997), contributing to the bacterium’s role in the development of periodontal disease (Lamont et al., 1994). Also, in fungal-bacterial communication AgI/II proteins play a pivotal role; specifically, this includes the interaction between *S. gordonii* SspB and the *C. albicans* hyphal cell wall protein Als3, which is relevant to biofilm formation (Silverman et al., 2010). Furthermore, AgI/II from *S. mutans* was shown to be important for the incorporation of *C. albicans* into a dual-species *S. mutans*-*C. albicans* biofilm and also required for increased acid production. Notably, this interaction is independent of the known streptococcal Als1 and Als3 receptors of *C. albicans* (Yang et al., 2018).

A group of streptococcal serine-rich repeat (Srr) glycoproteins can bind to sialoglycans present on salivary mucin MG2/MUC7, platelet glycoproteins (Takamatsu et al., 2005; Bensing et al., 2016) and polymorphonuclear neutrophils (PMNs) (Ruhl et al., 2000), and induce maturation and activation of DCs (Ko et al., 2017). Among these Srr glycoproteins are Gsp and Hsa of *S. gordonii* (Takahashi et al., 2002; Urano-Tashiro et al., 2016), the major fimbrial subunit Fap1 of *S. parasanguinis* (Wu et al., 2007) and *S. oralis* (Singh et al., 2017), SrpA, SrpB and SrpC of *S. salivarius* (Couvigny et al., 2017) and SrpA of *Streptococcus cristatus* (Handley et al., 2005) and *S. sanguinis* (Plummer et al., 2005; Deng et al., 2014). The interaction of these Sia-binding proteins with platelets may contribute to the pathogenesis of IE and potentially renders oral streptococci more virulent (Deng et al., 2014; Bensing et al., 2016).

*S. gordonii* additionally expresses short fibrils constituted by the CshA polypeptide on its cell surface (McNab et al., 1996). CshA as well as CshA-like fibrils present on the cell surface of other MGS such as *S. oralis* and *S. sanguinis* bind to other bacteria as well as salivary proteins (Fachon-Kalweit et al., 1985; Handley et al., 1987; Weerkamp et al., 1987; Ray et al., 1999) and immobilized fibronectin (McNab et al., 1996), thereby contributing to the establishment of the bacteria in the multispecies biofilms and colonization of various sites within the host. Other structures that oral streptococci are able to adhere to include glucan, collagen, plasminogen, laminin, and fibrinogen (Nobbs et al., 2009).

## 6 Streptococcal cell wall-associated virulence factors

### 6.1 AgI/II adhesin protein family

AgI/II adhesins are widely distributed among oral streptococci and constitute an important factor in the bacteria’s pathogenicity and ability to colonize oral sites (Jenkinson and Demuth, 1997) (see 5.). The AgI/II proteins consist of several structural regions, with the intertwined A- and P-regions presenting the globular, less conserved V-domain on the cell surface for ligand interaction (Manzer et al., 2020). *Viridans* streptococci reportedly induce the expression of proinflammatory cytokines IL-8 and IL-6 in endothelial and IL-8 in epithelial cells; in the case of *S. mutans* OMZ175, this is facilitated through the binding of AgI/II (as well as rhamnose-glucose polysaccharide (RGP) – see 7.3.) to glycoproteins present at the host cell surface through lectin-type interactions (Vernier et al., 1996). IL-8 stimulation occurs through binding of AgI/II to α5ß1 integrin and subsequent activation of MAPK signaling (Al-Okla et al., 1999). In primary human coronary artery endothelial cells, cellular binding of AgI/II was furthermore shown to stimulate the expression of the adhesion molecules E-selectin, ICAM-1 and VCAM-1 and thereby stimulate trans-endothelial migration of neutrophils and contribute to the exacerbation of inflammatory responses to the bacterial burden (Vernier-Georgenthum et al., 1998). Also, binding of AgI/II to monocytes occurs *via* a lectin-type interaction with Sia and fucose residues exposed on host cell surface-glycoproteins and results in an increased production of the proinflammatory cytokines TNF-α, Il-1β and Il-6 (Soell et al., 1994; Chatenay-Rivauday et al., 1998, 2000). Induction of pro-inflammatory cytokines in synovial cells suggests that AgI/II might even play a role in rheumatic disease, its initiation and perpetuation (Gourieux et al., 2001). Stimulation of TNF-α in THP-1 cells by the extended V-region of AgI/II was observed not only for *S. mutans* strains, but also for *S. gordonii* and the SAG members *S. anginosus*, *Streptococcus intermedius* and *Streptococcus constellatus*, highlighting the universal role the AgI/II protein family plays in streptococcal adhesion and virulence (Chatenay-Rivauday et al., 2000).

### 6.2 Cnm cell surface glycoprotein

Another factor contributing to *S. mutans’* success not only within but also outside its ecological niche is the cell surface glycoprotein Cnm which exhibits collagen- and laminin-binding capability. Cnm is present in 10-20% of all isolated strains of *S. mutans* (Nakano et al., 2010) and was most extensively studied in the S. *mutans* strain OMZ175. Cnm was found to be vital for the invasion of human coronary artery endothelial cells (HCAEC) and virulence in a *Galleria mellonella* infection model (Abranches et al., 2011; Aviles-Reyes et al., 2014). In the oral cavity, Cnm promotes *S. mutans’* invasion of oral keratinocytes and fibroblasts and enhances binding of the bacterium to collagenated surfaces, as well as dentin and root tissues contributing to its cariogenicity (Miller et al., 2015). Furthermore, Cnm was found to influence bacterial cell permeability and therefore might play a role in the susceptibility to antimicrobial agents (Naka et al., 2022).

Together with AgI/II, Cnm plays a crucial role in the aggravation of non-alcoholic steatohepatitis (NASH) (Naka et al., 2014, 2016, 2018); it is directly involved in and a potential risk factor for hemorrhagic stroke (Nakano et al., 2011) and IE (Nomura et al., 2013) and therefore can be considered as an indispensable virulence factor in the onset and progression of systemic diseases such as IE elicited by Cnm-positive *S. mutans* strains. Cnm is most frequently found in strains with serotype *f* (see 7.3.), such as *S. mutans* OMZ 175, which in contrast to serotype *c* strains, is not most common in dental plaque, but has been specifically implicated in the pathogenesis of systemic disease (Abranches et al., 2011).

### 6.3 Rhamnose-glucose polysaccharide

*S. mutans* decorates its cell surface with a rhamnose-glucose polysaccharide (RGP), a major cell wall antigen whose composition determines the strain serotype (*c*, *e*, *f*, and *k*) and which has distinct functions in cell division and morphology (Rijn and Bleiweis, 1973; Nakano and Ooshima, 2009; De et al., 2017). Also, in colonization of tooth surfaces - as well as heart muscle and kidney tissues - serotype *f* RGP plays a role as a putative adhesin (Stinson et al., 1980; Engels-Deutsch et al., 2003). The RGP’s contribution to adhesion was also demonstrated for THP-1, dental pulp and periodontal ligament cells, which upregulated the production of IL-6 and IL-8 in response to bacterial challenge dependent on the presence of RGP as well as AgI/II on the cell surface (Engels-Deutsch et al., 2003). RGP binds to human monocytes in a CD14-dependent manner and induces the release of TNF-α and other pro-inflammatory cytokines (Benabdelmoumene et al., 1991; Soell et al., 1995) as well as the up-regulation of RFc γ (Benabdelmoumene et al., 1991). RGP derived from *S. mutans* OMZ175 binds epithelial as well as endothelial cells in a dose-dependent manner and stimulates the release of IL-8 and IL-6 (Vernier et al., 1996). In rat model studies, RGP was found to render *S. mutans* more virulent in the induction of IE (Nagata et al., 2006) and stimulate nitric oxide synthase activity linking it to the genesis of septic shock induced by Gram-positive bacteria (Martin et al., 1997). Furthermore, the hydrophilic nature of RGP might play a role in the resistance of *S. mutans* to phagocytosis by PMNs and thereby contribute to the bacteria’s defiance of immune response mechanisms and survival within the host (Tsuda et al., 2000).

### 6.4 Lipoteichoic acid

In Gram-positive bacteria, teichoic acids – wall teichoic acids (WTA) anchored to the cell wall peptidoglycan and lipoteichoic acids (LTA) anchored to membrane glycolipids - constitute a major part of the cell envelope and play vital roles in bacterial physiology, surface attachment, as well as interspecies and host interactions (Silhavy et al., 2010). Depending on the chemical structure, LTAs can be classified into five types (I-V) (Percy and Gründling, 2014). Type I, present *e.g.*, in *S. mutans* and *S. gordonii*, is comprised of repeating polyglycerolphosphate units coupled with D-alanine and glucose (Lima et al., 2019); type II LTA as present in *S. oralis*, is composed of polyribitolphosphate repeating units (Hong et al., 2014a).

In the context of cariogenesis, LTA plays a vital role in conferring adhesive properties to bacterial cells and hydration of the cariogenic plaque in concert with streptococcal derived glucan (Rølla et al., 2009). When provided with high amounts of sucrose, *S. mutans* upregulates not only its glucan production, but also produces higher amounts of LTA (Rølla et al., 2009). *S. mutans* LTA furthermore induces apoptosis in pulpal cells from deciduous teeth indicating its involvement in the development to pulpitis (Wang et al., 2001), which has been reviewed elsewhere (Hahn and Liewehr, 2007b). In monocytic, dental pulp and periodontal ligament cells, *S. mutans* LTA is a less potent inducer of proinflammatory cytokines than RGP and AgI/II (Engels-Deutsch et al., 2003). In a murine macrophage cell line, LTA from both *S. mutans* and *S. sanguinis* induced the production of TNF-α and release of nitric oxide (NO) potentially contributing to septic shock elicited by these bacteria in immunocompromised patients (English et al., 1996; Hong et al., 2014a).

When analyzing *S. mutans* LTA-binding proteins in saliva of caries-free and caries-active subjects, Hong and coworkers found differences in protein profiles dependent on the health status, with histone H4, neutrophil defensin-1 and profilin-1 being associated with health, and cystatins and lysozyme, among others, predominantly present in saliva from caries patients (Hong et al., 2014b). In the case *S. sanguinis*, LTA was shown to antagonize recognition of lipopolysaccharide (LPS) on gingival fibroblasts in a CD14-dependent manner, thereby potentially dampening the cells’ immune response to Gram-negative periodontal pathogens and contributing to commensalism (Sugawara et al., 1999). LTA in contrast to LPS also stimulates expression of hepatocyte growth factor/scatter factor in gingival epithelial cells (Sugiyama et al., 1996). When stimulated with an LTA-deficient strain of *S. gordonii*, human dendritic cells reacted with a stronger immune response, *i.e.*, increased phagocytic activity and production of proinflammatory markers and T-cell activation, than those treated with the corresponding parent wild-type strain, indicating an immune evasive role of LTA (Kim et al., 2023). In this context it is interesting to note, that *S. gordonii* cell wall lipoproteins, but not LTA have been shown to stimulate IL-8 production in periodontal ligament cells in a TLR2-dependent fashion (Kim et al., 2017; Park et al., 2020) and, in the case of lipoprotein PpiA, to suppress phagocytosis by macrophages (Cho et al., 2013).

### 6.5 Glycosyltransferases

Colonization of the oral cavity by oral streptococci depends on the production of extracellular glucans that to a large part make up the ECM of streptococcal biofilms (Nobbs et al., 2015; Bowen et al., 2018). *S. mutans* produces several dietary sucrose-hydrolyzing enzymes producing fructose and glucose, which are the building blocks of α-1,2- and β-1,6-linked glucans formed by the subsequent action of secreted (GtfD) and cell-wall associated (GtfB, GtfC) glucosyltransferases (Gtfs) (Nobbs et al., 2015; Bowen et al., 2018). In cariogenic biofilms, these sugars contribute to the acidification of the biofilm microenvironment and the shift to a dysbiotic microbiota, since these are catabolized along fermentative pathways leading to acid production (Bowen et al., 2018; Lamont et al., 2018). The secretion and action of Gtfs is not a specific trait of *S. mutans*, in which these enzymes have been most extensively studied; many oral streptococci employ Gtfs for colonization, adhesion, cohesion, and interbacterial interactions (Nobbs et al., 2009; Abranches et al., 2018). GtfB, GftC and GtfD adhere to the salivary pellicle on the tooth surface as well as to other microorganisms including bacteria and *Candida albicans*, essentially converting them to glucan producers contributing to the build-up of EPS and biofilm formation (Falsetta et al., 2014; Bowen, 2016). Furthermore, glucan binding proteins (Gbps) expressed by MS are prerequisites for successful biofilm formation, facilitating binding to dextrans and accumulation of MS in the biofilms (Nobbs et al., 2009). Streptococcal Gtfs can be regarded as immunogenic; a humoral immune response with anti-Gtf-specific immunoglobulin G (IgG) in serum or IgA in saliva naturally occurs in human populations (Chia et al., 1997). A Gtf-inhibitory factor (GIF) was found as an innate defense factor present in human saliva, specifically binding to the glucan binding domain of Gtfs and inhibiting Gtf function (Jespersgaard et al., 2002). GtfC and GtfD influence T-cell proliferation and modulate the immune response by monocytes, with a higher response elicited by GtfD than GtfC (Chia et al., 2001). *S. mutans* Gtfs furthermore robustly induce the production of IL-6 by T-cells *in vitro* as well as *in vivo* in a experimental rat model of endocarditis – pinpointing their contribution to disease development outside the oral cavity, since IL-6 levels were found to be elevated in patients with IE (Chia et al., 2001). In the same experimental model, it was also shown that *S. mutans* Gtfs induce the production of IL-6 in endothelial cells in infected heart valves, specifically during the acute stage of infection (Shun et al., 2005). In *S. gordonii*, a Gtf contributes to the bacterium’s capability to cause IE, being a prerequisite for the adhesion to endothelial as well as epithelial cells *in vitro* (Vacca-Smith et al., 1994).

### 6.6 Nucleases

In the resolution of bacterial infection, neutrophils play a pivotal role, clearing and killing bacterial invaders through phagocytosis, granule release, oxidative burst, and neutrophil extracellular trap (NET) formation – the release of chromatin fibers carrying antimicrobial peptides (AMPs) ready for bacterial killing and prevention of bacterial spreading (Scott and Krauss, 2012; Chapple et al., 2023). In *S. sanguinis*, a streptococcal wall-anchored nuclease (SWAN) confers resistance to NET killing by digesting released NET DNA (Morita et al., 2014). Another *S. mutans* nuclease - DeoC - enables the bacteria to escape NETs and facilitates biofilm dispersal (Liu et al., 2017). Lacking the LPxTG sorting motif for cell wall anchoring, DeoC most likely acts during late stages of biofilm growth when cell autolysis and dispersal occur and aids in bacterial escape of entrapment in NETs by chromatin degradation (Liu et al., 2017).

## 7 Streptococcal small molecules at the host immune interface

### 7.1 Hydrogen peroxide

One characteristic trait of oral streptococci that has been getting attention for its relevance in the regulation of immune responses is the production of hydrogen peroxide as a by-product of aerobic metabolism (Zhu and Kreth, 2012). Through the production of H_2_O_2_, oral streptococci like MGS inhibit other potentially pathogenic species, among them *S. mutans*, and thereby gain a competitive advantage over these species (Kreth et al., 2008). By triggering the release of eDNA, H_2_O_2_ production additionally facilitates the exchange of genetic material between the bacteria (Kreth et al., 2009). In the interaction with the host, H_2_O_2_ production shows the potential to be a vital contributor to disease pathogenesis. Bacterial species such as *S. sanguinis* and *S. oralis* induce the formation of foam cells, contributing to atherosclerosis, and cause lysosome dysfunction and cell death in macrophages as well as in epithelial cells through the action of ROS, *i.e.*, the release of H_2_O_2_ (Okahashi et al., 2011, 2013, 2014, 2016). Microarray studies of macrophages infected with *S. oralis* wild-type and a corresponding streptococcal pyruvate oxidase (*spx*)-deletion mutant deficient in H_2_O_2_ production showed that H_2_O_2_ suppresses the expression of proinflammatory mediators, especially NF-κB signaling, and cellular stress responses (Matsushima et al., 2017). Pyruvate oxidase SpxB catalyzes the conversion of inorganic phosphate and pyruvate to acetyl phosphate, carbon dioxide and H_2_O_2_ (Chen et al., 2011). Notably, SpxB is distinct from the *S. mutans* transcriptional regulator Spx, encoding two homologues, SpxA1 and SpxA2, involved in oxidative stress response and regulation of genes involved in cell division and cell envelope biosynthesis, respectively (Baker et al., 2020; Ganguly et al., 2020). *S. oralis* was furthermore found to inhibit the activation of inflammasomes through the release of H_2_O_2_ corroborating its contribution to the establishment and persistence of the oral commensal within the oral cavity and even in bloodstream infections (Erttmann and Gekara, 2019). In the context of periodontal disease, SpxB of *S. oralis* and *S. mitis* plays a vital role in enabling bacterial colonization and host homeostasis by inhibiting NF-κB signaling through the activation of nuclear factor erythroid 2-related factor 2 (Nrf2) (Tang et al., 2022). Also, through the induction of cell death in periodontal ligament cells (PDLs) by H_2_O_2_ production, *S. gordonii*, *S. mitis*, *S. sanguinis* and *S. sobrinus* play their part in the development of apical periodontitis (Park et al., 2021). In *S. sanguinis*, Spx was a prerequisite for bacterial survival in blood and an *spx*-deletion mutant failed to induce cell death and NET formation in neutrophils (Sumioka et al., 2017). Through the release of H_2_O_2_, oral streptococci can additionally dampen the proinflammatory immune response to LPS and *F. nucleatum* thereby influencing overall plaque development (Tang et al., 2022).

### 7.2 Secondary metabolites

Genome mining studies have revealed an abundance of bacterial biosynthetic gene clusters (BGCs) in the oral microbiome producing small molecules and secondary metabolites that can function as signaling molecules and serve as language of interbacterial, interspecies and interkingdom communication (Donia and Fischbach, 2015; Liu et al., 2016; Aleti et al., 2019). Among these secondary metabolites are mutanobactins, mutanamide and mutanofactins - lipopeptides produced by *S. mutans* along a non-ribosomal peptide synthetase–polyketide synthase (NRPS–PKS) assembly line (Wu et al., 2010; Zvanych et al., 2015; Li et al., 2021). Mutanobactins act as interkingdom signaling molecules by blunting hyphae formation and inhibiting biofilm formation by the opportunistic pathogen *C. albicans* and as interspecies communicator by inhibiting planktonic growth of other oral bacteria (Joyner et al., 2010; Wu et al., 2010; Wang et al., 2012; Pultar et al., 2021). Given that, co-infection with *S. mutans* and *C. albicans* not only resulted in a higher bacterial-fungal burden, but also increased biofilm virulence and led to more severe carious lesion in a rat model (Falsetta et al., 2014), hyphae blunting by mutanobactin represents only a small facet of this highly complex interkingdom interaction and the implications of this process *in vivo* have yet to be determined. Interestingly, Zvanych et al. attested to the mutanobactins also immunomodulatory properties, since in a murine macrophage cell line pre-stimulated with LPS, mutanobactin B upregulated IL-6 and IL-12 and downregulated MCP-1, G-CSF and TNF-α production (Zvanych et al., 2015). Another small molecule produced by *S. mutans* with the potential to inhibit competing oral bacteria, is the tetramic acid mutanocyclin (Tang et al., 2020). In a Matrigel plug assay, mutanocyclin was found to inhibit CD45^+^ leukocyte infiltration thereby exerting an anti-inflammatory role (Hao et al., 2019).

### 7.3 Competence stimulating peptide CSP-1

For the development of microbial communities, quorum sensing (QS) is an indispensable mechanism of bacteria to regulate inter- and intra-species interactions*. S. mutans* utilizes multiple QS systems to regulate biofilm formation, transfer of genetic material and stress tolerance responses; one of these systems relies on the competence stimulating peptide (CSP) encoded by the *comCDE* gene locus (Bernabè et al., 2022). Medapati and coworkers recently explored the possibility that *S. mutans* CSPs might be recognized by bitter taste receptors (T2Rs) - G-protein coupled receptors (GPCRs) involved in taste chemosensation and recognition of bacterial QS molecules – thereby participating in innate immune responses to bacterial colonization (Medapati et al., 2021). The researchers demonstrated binding of CSP-1 to T2R14 expressed in gingival epithelial cells (GECs) and mediating activation of IL-6, IL-8 and TNF-α production, thus, identifying a novel mechanism of host-QS interaction and a potential target for therapeutic intervention (Medapati et al., 2021).

## 8 Effect of oral streptococci on the host immune response to other oral bacteria

In multispecies communities, interactions between species – through synergistic or antagonistic effects - can be a decisive factor when it comes to survival and persistence within the host. In the context of periodontal disease, for instance, *P. gingivalis* can subvert the host immune responses through numerous mechanisms, enabling other members of the biofilm community to escape immune surveillance and increase in cell numbers (Hajishengallis and Lamont, 2014). *P. gingivalis* and other late biofilm colonizers require the presence of streptococci and *F. nucleatum* in order to establish themselves within the biofilm community profiting from reduced oxygen tension and the provision of metabolites and nutrients by the antecedent colonizers (Kuboniwa and Lamont, 2010).

### 8.1 MGS and their interaction partners at the immune interface

Due to their co-adhesion with *P. gingivalis*, MGS can be classified as accessory pathogens and contribute to the formation of pathogenic oral communities (Whitmore and Lamont, 2011). The formation of heterotypic communities between the different species has been extensively studied for *S. gordonii* and *S. oralis* with *P. gingivalis*. Cohesion occurs through the binding between *P. gingivalis* fimbriae Mfa1 and FimA to the streptococcal surface antigens SspA/B and surface-expressed glyceraldehyde-3-phosphate dehydrogenase (GAPDH), respectively (Bergmann et al., 2004; Kuboniwa and Lamont, 2010). Upon binding, a signaling cascade is triggered in *P. gingivalis*, leading to the suppression of Mfa1 production and the expression of a protein tyrosine phosphatase Ltp1 which regulates protease activity and thereby influences the bacterium’s pathogenic potential (Maeda et al., 2008; Kuboniwa and Lamont, 2010; Hajishengallis and Lamont, 2014).

Another species that *S. gordonii* is frequently associated with is *F. nucleatum* (Mutha et al., 2018). Co-aggregation not only results in transcriptional changes within both bacteria, but also affects their survival within macrophages and decreases the expression of pro-inflammatory cytokines and thereby influences bacterial virulence and host persistence (Liu et al., 2021). Also the commensal *S. cristatus* dampens the response to *F. nucleatum* infection by inhibiting NF-κB and IL-8 production in oral epithelial cells (Zhang et al., 2008 *;* Zhang et al., 2011).

Co-infection studies with *S. oralis* and *C. albicans* demonstrated that in an oral thrush mouse model, *C. albicans* augments streptococcal colonization and that, in turn, *S. oralis* contributes to the exacerbation of the immune response through TLR2 signaling and neutrophil recruitment causing more severe lesions than the opportunistic fungal pathogen alone (Xu et al., 2014). In epithelial tissues, the synergy between the two bacteria caused upregulation of pro-inflammatory markers (Martorano-Fernandes et al., 2023) and the disruption of epithelial barrier integrity through activation of calpain 1, a calcium ion–dependent cysteine protease specifically cleaving E-cadherin (Xu et al., 2016). Interaction with the allegedly harmless commensal *S. oralis* therefore seems to be a key contributor to *C. albicans* virulence and tissue invasion.

When oral keratinocytes were precultured with *S. cristatus*, *S. salivarius*, *S. mitis* or *S. sanguinis*, the presence of the oral streptococci inhibited keratinocyte colonization by *A. actinomycetemcomitans*, with *S. sanguinis* exerting the strongest inhibitory effect (Teughels et al., 2007; Sliepen et al., 2009b). In a similar experiment, adhesion of periodontal pathogens, including *P. gingivalis*, *Prevotella intermedia* and *A. actinomycetemcomitans*, to the surface of a parallel plate flow chamber was found to be reduced in the presence of a similar set of commensal streptococci, identifying antagonistic functions that could render these bacteria suitable candidates as probiotics in the prevention of periodontal disease recurrence (Van Hoogmoed et al., 2008). Different strains of *S. salivarius* alone reduced the secretion of IL-6 and IL-8 by gingival fibroblasts, which is usually induced by *P. gingivalis*, *A. actinomycetemcomitans* and *F. nucleatum*, both in co-infection studies and when fibroblasts were pre-treated with the commensal before pathogen stimulation (MacDonald et al., 2021). Notably, the tested *S. salivarius* strains did not elicit enhanced pro-inflammatory cytokine secretion or microbiome alterations in healthy volunteers when administered in the form of a chewing gum and therefore might be interesting candidates for a probiotic therapy of periodontal disease (MacDonald et al., 2021).

The capacity of oral streptococci to modulate the tissue response to oral pathogens has also been discussed in the context of probiotic treatment of oral biofilm disease. Detected solely in samples from caries- and periodontitis-free patients, the rather recently discovered *Streptococcus dentisani* might be a potential candidate probiotic agent (Esteban-Fernández et al., 2019 *;* Ferrer et al., 2020 *). In vitro* studies showed that *S. dentisani* induced secretion of anti-inflammatory cytokine IL-10 in gingival fibroblasts, reduced the pro-inflammatory response to *P. gingivalis* and *F. nucleatum*, and inhibited growth as well as colonization of fibroblasts by these Gram-negative bacteria (Esteban-Fernández et al., 2019). This highlights the role of oral streptococci in maintaining oral health through their interaction with the host immune system and exertion of antagonistic effects towards pathogenic species.

## 9 Conclusions and future perspectives

**Figure 2:**
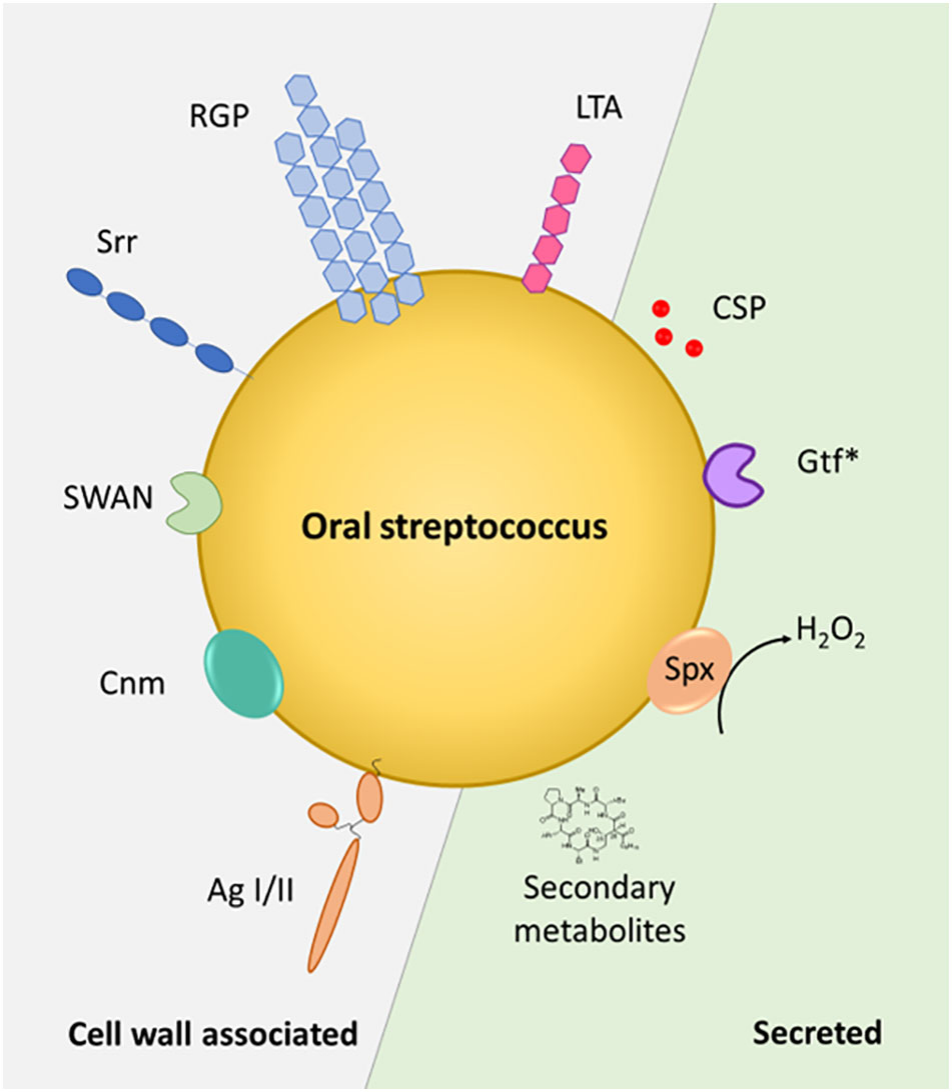
Streptococcal factors at the host interface. Cell wall-associated and secreted factors are depicted in the schematic representation of an oral *Streptococcus* sp. Gtf*, glycosyltransferases - GtfB and GtfC are cell wall associated, GtfD is secreted – Spx – streptococcal pyruvate oxidase. AgI/II – antigen I/II, Srr – serine-rich repeat protein, CSP – competence stimulating peptide, SWAN – cell-wall associated nuclease, RGP – rhamnose-glucose polymer, LTA – lipoteichoic acid, Cnm - surface glycoprotein Cnm with collagen- and laminin- binding capability.

Streptococci have a profound and life-long presence in humans, being the first colonizers in the oral cavity after birth and continuing to shape the intricate microbiota within our mouth throughout our life. *Streptococcus* species are commensal inhabitants, influencing the development of a properly functioning immune system and acting as barriers against the colonization of harmful bacteria – functions that are crucial for maintaining oral health and preventing disease. On the other hand, when things go awry, streptococci can become detrimental, potentially leading to serious infections and health complications, including infective endocarditis, purulent infections, brain hemorrhage, intestinal inflammation, autoimmune diseases, and bacteremia (Yumoto et al., 2019). A key characteristic in these processes is the ability of these species to adhere to numerous substrates present on host cell surfaces and within the dental pellicle and to form biofilms (**Figure 1**). This is facilitated through specialized surface proteins such as AgI/I, Srr proteins, Cnm and RGP formation through the action of Gtfs (Nobbs et al., 2009; Nakano et al., 2011) (**Figure 2**). Also, in the interaction with the host immune system these factors play a vital role by modulating the induction of proinflammatory immune responses, bacterial cell invasion and adhesion. In the context of therapeutic approaches to dental caries, the most researched virulence antigens of *S. mutans* are AgI/II, Gtfs and Gbps (Patel, 2020). RGP and LTA as constituents of the cell wall act as potent inducers of pro-inflammatory cytokine production and confer resistance to phagocytosis (Tsuda et al., 2000; Engels-Deutsch et al., 2003). In *S. sanguis* and *S. mutans*, SWAN confers resistance to NET killing and therefore enables these bacteria to evade immune defense mechanisms (Liu et al., 2017; Kim et al., 2023). Through the production of H_2_O_2_ commensal oral streptococci antagonize pathogens such as *S. mutans* or *A. actinomycetemcomitans* (Baty et al., 2022). While commensal streptococci such as *S. mitis* or *S. salivarius* can downregulate cytokine production in response to challenge with periodontal pathogens and thereby counteract gingival inflammation, others such as *S. gordonii* can be regarded as accessory pathogens and support colonization by periodontal pathogens.

For vaccine development and caries treatment, the identification of novel streptococcal virulence factors is a central prerequisite. Understanding virulence mechanisms and interactions within the microbial networks in oral biofilms will help to develop targeted therapeutic or preventative measures to decrease the burden of oral diseases. Promising results achieved with the use of health-associated streptococci as probiotics such as *S. salivarius* or *S. dentisani* highlight the importance of oral streptococci in oral microbial interaction networks (Ferrer et al., 2020; MacDonald et al., 2021). Streptococci are essential in the maintenance of homeostasis and controlling the composition of the oral microbiota, but their role in regulating host-microbiome interaction is still to be unraveled. The host response regulation exerted by these bacterial species could be a key factor and future studies on this topic will allow a better understanding of oral streptococci and their roles in oral health and disease.

## Author contributions

SB: Conceptualization, Formal Analysis, Writing – original draft, Writing – review & editing. FH-M: Formal Analysis, Writing – original draft, Writing – review & editing, Funding acquisition. OA: Writing – original draft, Writing – review & editing, Funding acquisition. CS: Writing – original draft, Writing – review & editing, Funding acquisition, Conceptualization, Formal Analysis, Project administration, Supervision.

## References

[B1] Aas J. A. Paster B. J. Stokes L. N. Olsen I. Dewhirst F. E. (2005). Defining the normal bacterial flora of the oral cavity. J. Clin. Microbiol. 43, 5721–5732. doi: 10.1128/JCM.43.11.5721-5732.2005 16272510 PMC1287824

[B2] Abranches J. Miller J. H. Martinez A. R. Simpson-Haidaris P. J. Burne R. A. Lemos J. A. (2011). The collagen-binding protein Cnm is required for *Streptococcus mutans* adherence to and intracellular invasion of human coronary artery endothelial cells. Infect.Immun. 79, 2277–2284. doi: 10.1128/IAI.00767-10 21422186 PMC3125845

[B3] Abranches J. Zeng L. Kajfasz J. K. Palmer S. R. Chakraborty B. Wen Z. T.. (2018). Biology of oral streptococci. Microbiol. Spectr. 6, GPP3-0042-2018. doi: 10.1128/microbiolspec.GPP3-0042-2018 PMC6287261 30338752

[B4] Abusleme L. Dupuy A. K. Dutzan N. Silva N. Burleson J. A. Strausbaugh L. D.. (2013). The subgingival microbiome in health and periodontitis and its relationship with community biomass and inflammation. ISME J. 7, 1016–1025. doi: 10.1038/ismej.2012.174 23303375 PMC3635234

[B5] Aleti G. Baker J. L. Tang X. Alvarez R. Dinis M. Tran N. C.. (2019). Identification of the bacterial biosynthetic gene clusters of the oral microbiome illuminates the unexplored social language of bacteria during health and disease. mBio 10, e00321-19. doi: 10.1128/mBio.00321-19 30992349 PMC6469967

[B6] Al-Okla S. Chatenay-Rivauday C. Klein J.-P. Wachsmann D. (1999). Involvement of α5β1 integrins in interleukin 8 production induced by oral viridans streptococcal protein I/IIf in cultured endothelial cells. Cell. Microbiol. 1, 157–168. doi: 10.1046/j.1462-5822.1999.00016.x 11207549

[B7] Alves L. A. de Carli T. R. Harth-Chu E. N. Mariano F. S. Höfling J. F. Stipp R. N.. (2019). Oral streptococci show diversity in resistance to complement immunity. J. Med. Microbiol. 68, 600–608. doi: 10.1099/jmm.0.000955 30843785

[B8] Alves L. A. Nomura R. Mariano F. S. Harth-Chu E. N. Stipp R. N. Nakano K.. (2016). CovR regulates *Streptococcus mutans* susceptibility to complement immunity and survival in blood. Infect. Immun. 84, 3206–3219. doi: 10.1128/IAI.00406-16 27572331 PMC5067753

[B9] Aviles-Reyes A. Miller J. H. Simpson-Haidaris P. J. Hagen F. K. Abranches J. Lemos J. A. (2014). Modification of *Streptococcus mutans* Cnm by PgfS contributes to adhesion, endothelial cell invasion, and virulence. J. Bacteriol. 196, 2789–2797. doi: 10.1128/JB.01783-14 24837294 PMC4135665

[B10] Baker J. L. Saputo S. Faustoferri R. C. Quivey R. G. Jr. (2020). *Streptococcus mutans* SpxA2 relays the signal of cell envelope stress from LiaR to effectors that maintain cell wall and membrane homeostasis. Mol. Oral Microbiol. 35, 118–128. doi: 10.1111/omi.12282 32043713 PMC7202993

[B11] Baty J. J. Stoner S. N. Scoffield J. A. (2022). Oral commensal streptococci: Gatekeepers of the oral cavity. J. Bacteriol. 204, e0025722. doi: 10.1128/jb.00257-22 36286512 PMC9664950

[B12] Benabdelmoumene S. Dumont S. Petit C. Poindron P. Wachsmann D. Klein J. P. (1991). Activation of human monocytes by *Streptococcus mutans* serotype f polysaccharide: immunoglobulin G Fc receptor expression and tumor necrosis factor and interleukin-1 production. Infect. Immun. 59, 3261–3266. doi: 10.1128/iai.59.9.3261-3266.1991 1831797 PMC258161

[B13] Bensing B. A. Khedri Z. Deng L. Yu H. Prakobphol A. Fisher S. J.. (2016). Novel aspects of sialoglycan recognition by the Siglec-like domains of streptococcal SRR glycoproteins. Glycobiology 26, 1222–1234. doi: 10.1093/glycob/cww042 27037304 PMC6086536

[B14] Bergmann S. Rohde M. Hammerschmidt S. (2004). Glyceraldehyde-3-phosphate dehydrogenase of *Streptococcus pneumoniae* is a surface-displayed plasminogen-binding protein. Infect. Immun. 72, 2416–2419. doi: 10.1128/IAI.72.4.2416-2419.2004 15039372 PMC375162

[B15] Bernabè G. Pauletto A. Zamuner A. Cassari L. Castagliuolo I. Brun P.. (2022). Exploiting conserved quorum sensing signals in *Streptococcus mutans* and *Streptococcus pneumoniae* . Microorganisms 10, 2386. doi: 10.3390/microorganisms10122386 36557639 PMC9785397

[B16] Bloch S. Tomek M. B. Friedrich V. Messner P. Schäffer C. (2019). Nonulosonic acids contribute to the pathogenicity of the oral bacterium *Tannerella forsythia* . Interface Focus 9, 20180064. doi: 10.1098/rsfs.2018.0064 30842870 PMC6388019

[B17] Bloch S. Zwicker S. Bostanci N. Sjöling A. Bostrom E. A. Belibasakis G. N.. (2018). Immune response profiling of primary monocytes and oral keratinocytes to different *Tannerella forsythia* strains and their cell surface mutants. Mol. Oral Microbiol. 33, 155–167. doi: 10.1111/omi.12208 29235255

[B18] Bowen W. H. (2016). Dental caries - not just holes in teeth! A perspective. Mol. Oral Microbiol. 31, 228–233. doi: 10.1111/omi.12132 26343264

[B19] Bowen W. H. Burne R. A. Wu H. Koo H. (2018). Oral biofilms: pathogens, matrix, and polymicrobial interactions in microenvironments. Trends Microbiol. 26, 229–242. doi: 10.1016/j.tim.2017.09.008 29097091 PMC5834367

[B20] Brady L. J. Maddocks S. E. Larson M. R. Forsgren N. Persson K. Deivanayagam C. C.. (2010). The changing faces of *Streptococcus* antigen I/II polypeptide family adhesins. Mol. Microbiol. 77, 276–286. doi: 10.1111/j.1365-2958.2010.07212.x 20497507 PMC2909373

[B21] Chamat-Hedemand S. Dahl A. Østergaard L. Arpi M. Fosbøl E. Boel J.. (2020). Prevalence of infective endocarditis in streptococcal bloodstream infections is dependent on streptococcal species. Circulation 142, 720–730. doi: 10.1161/CIRCULATIONAHA.120.046723 32580572

[B22] Chapple I. L. C. Hirschfeld J. Kantarci A. Wilensky A. Shapira L. (2023). The role of the host-neutrophil biology. Periodontol. 2000, 1–47. doi: 10.1111/prd.12490 37199393

[B23] Chatenay-Rivauday C. Yamodo I. Sciotti M. A. Ogier J. A. Klein J. P. (1998). The A and the extended V N-terminal regions of streptococcal protein I/IIf mediate the production of tumour necrosis factor alpha in the monocyte cell line THP-1. Mol. Microbiol. 29, 39–48. doi: 10.1046/j.1365-2958.1998.00881.x 9701801

[B24] Chatenay-Rivauday C. Yamodo I. Sciotti M. A. Troffer-Charlier N. Klein J. P. Ogier J. A. (2000). TNF-alpha release by monocytic THP-1 cells through cross-linking of the extended V-region of the oral streptococcal protein I/II. J. Leukoc. Biol. 67, 81–89. doi: 10.1002/jlb.67.1.81 10648001

[B25] Chávez de Paz L. Svensäter G. Dahlén G. Bergenholtz G. (2005). Streptococci from root canals in teeth with apical periodontitis receiving endodontic treatment. Oral Surg. Oral Med. Oral Pathol. Oral Radiol. Endod. 100, 232–241. doi: 10.1016/j.tripleo.2004.10.008 16037782

[B26] Chen L. Ge X. Dou Y. Wang X. Patel J. R. Xu P. (2011). Identification of hydrogen peroxide production-related genes in *Streptococcus sanguinis* and their functional relationship with pyruvate oxidase. Microbiology 157, 13–20. doi: 10.1099/mic.0.039669-0 20847003 PMC3069532

[B27] Chia J.-S. Lin S.-W. Yang C.-S. Chen J.-Y. (1997). Antigenicity of a synthetic peptide from glucosyltransferases of *Streptococcus mutans* in humans. Infect. Immun. 65, 1126–1130. doi: 10.1128/iai.65.3.1126-1130.1997. 9038329 PMC175101

[B28] Chia J.-S. You C.-M. Hu C.-Y. Chiang B.-L. Chen J.-Y. (2001). Human T-cell responses to the glucosyltransferases of *Streptococcus mutans* . Clin. Diagn. Lab. Immunol. 8, 441–445. doi: 10.1128/CDLI.8.2.441-445.2001 11238236 PMC96077

[B29] Cho K. Arimoto T. Igarashi T. Yamamoto M. (2013). Involvement of lipoprotein PpiA of *Streptococcus gordonii* in evasion of phagocytosis by macrophages. Mol. Oral Microbiol. 28, 379–391. doi: 10.1111/omi.12031 23734737

[B30] Conrads G. de Soet J. J. Song L. Henne K. Sztajer H. Wagner-Döbler I.. (2014). Comparing the cariogenic species *Streptococcus sobrinus* and *Streptococcus mutans* on whole genome level. J. Oral Microbiol. 6, 26189. doi: 10.3402/jom.v6.26189 25475081 PMC4256546

[B31] Cosseau C. Devine D. A. Dullaghan E. Gardy J. L. Chikatamarla A. Gellatly S.. (2008). The commensal *Streptococcus salivarius* K12 downregulates the innate immune responses of human epithelial cells and promotes host-microbe homeostasis. Infect. Immun. 76, 4163–4175. doi: 10.1128/IAI.00188-08 18625732 PMC2519405

[B32] Couvigny B. Lapaque N. Rigottier-Gois L. Guillot A. Chat S. Meylheuc T.. (2017). Three glycosylated serine-rich repeat proteins play a pivotal role in adhesion and colonization of the pioneer commensal bacterium, *Streptococcus salivarius* . Environ. Microbiol. 19, 3579–3594. doi: 10.1111/1462-2920.13853 28695648

[B33] Daep C. A. Novak E. A. Lamont R. J. Demuth D. R. (2011). Structural dissection and in *vivo* effectiveness of a peptide inhibitor of *Porphyromonas gingivalis* adherence to *Streptococcus gordonii* . Infect. Immun. 79, 67–74. doi: 10.1128/IAI.00361-10 21041492 PMC3019905

[B34] De A. Liao S. Bitoun J. P. Roth R. Beatty W. L. Wu H.. (2017). Deficiency of RgpB causes major defects in cell division and biofilm formation, and deficiency of Lytr-Cpsa-Psr family proteins leads to accumulation of cell wall antigens in culture medium by *Streptococcus mutans* . Appl. Environ. Microbiol. 83, e00928-17. doi: 10.1128/aem.00928-17 28687645 PMC5561293

[B35] Deng L. Bensing B. A. Thamadilok S. Yu H. Lau K. Chen X.. (2014). Oral streptococci utilize a Siglec-like domain of serine-rich repeat adhesins to preferentially target platelet sialoglycans in human blood. PloS Pathog. 10, e1004540. doi: 10.1371/journal.ppat.1004540 25474103 PMC4256463

[B36] Diaz P. I. Chalmers N. I. Rickard A. H. Kong C. Milburn C. L. Palmer R. J. Jr.. (2006). Molecular characterization of subject-specific oral microflora during initial colonization of enamel. Appl. Environ. Microbiol. 72, 2837–2848. doi: 10.1128/AEM.72.4.2837-2848.2006 16597990 PMC1449052

[B37] Donia M. S. Fischbach M. A. (2015). Small molecules from the human microbiota. Science 349, 1254766. doi: 10.1126/science.1254766 26206939 PMC4641445

[B38] Eberhard J. Pietschmann R. Falk W. Jepsen S. Dommisch H. (2009). The immune response of oral epithelial cells induced by single-species and complex naturally formed biofilms. Oral Microbiol. Immunol. 24, 325–330. doi: 10.1111/j.1399-302X.2009.00518.x 19572896

[B39] Ebersole J. L. Dawson D. III Emecen-Huja P. Nagarajan R. Howard K. Grady M. E.. (2017). The periodontal war: microbes and immunity. Periodontol. 2000 75, 52–115. doi: 10.1111/prd.12222 28758303

[B40] Engels-Deutsch M. Pini A. Yamashita Y. Shibata Y. Haikel Y. Schöller-Guinard M.. (2003). Insertional inactivation of pac and *rmlB* genes reduces the release of tumor necrosis factor alpha, interleukin-6, and interleukin-8 induced by *Streptococcus mutans* in monocytic, dental pulp, and periodontal ligament cells. Infect. Immun. 71, 5169–5177. doi: 10.1128/IAI.71.9.5169-5177.2003 12933861 PMC187322

[B41] Engen S. A. Schreurs O. Petersen F. Blix I. J. S. Baekkevold E. S. Schenck K. (2018). The regulatory role of the oral commensal *Streptococcus mitis* on human monocytes. Scand. J. Immunol. 87, 80–87. doi: 10.1111/sji.12636 29194752

[B42] English B. K. Patrick C. C. Orlicek S. L. McCordic R. Shenep J. L. (1996). Lipoteichoic acid from viridans streptococci induces the production of tumor necrosis factor and nitric oxide by murine macrophages. J. Infect. Dis. 174, 1348–1351. doi: 10.1093/infdis/174.6.1348 8940232

[B43] Erttmann S. F. Gekara N. O. (2019). Hydrogen peroxide release by bacteria suppresses inflammasome-dependent innate immunity. Nat. Commun. 10, 3493. doi: 10.1038/s41467-019-11169-x 31375698 PMC6677825

[B44] Esteban-Fernández A. Ferrer M. D. Zorraquín-Peña I. López-López A. Moreno-Arribas M. V. Mira A. (2019). *In vitro* beneficial effects of *Streptococcus dentisani* as potential oral probiotic for periodontal diseases. J. Periodontol. 90, 1346–1355. doi: 10.1002/JPER.18-0751 31111495

[B45] Fachon-Kalweit S. Elder B. L. Fives-Taylor P. (1985). Antibodies that bind to fimbriae block adhesion of *Streptococcus sanguis* to saliva-coated hydroxyapatite. Infect. Immun. 48, 617–624. doi: 10.1128/iai.48.3.617-624.1985 2860066 PMC261206

[B46] Facklam R. (2002). What happened to the streptococci: overview of taxonomic and nomenclature changes. Clin. Microbiol. Rev. 15, 613–630. doi: 10.1128/CMR.15.4.613-630.2002 12364372 PMC126867

[B47] Falsetta M. L. Klein M. I. Colonne P. M. Scott-Anne K. Gregoire S. Pai C. H.. (2014). Symbiotic relationship between *Streptococcus mutans* and *Candida albicans* synergizes virulence of plaque biofilms in *vivo* . Infect. Immun. 82, 1968–1981. doi: 10.1128/IAI.00087-14 24566629 PMC3993459

[B48] Farges J.-C. Alliot-Licht B. Renard E. Ducret M. Gaudin A. Smith A. J.. (2015). Dental pulp defence and repair mechanisms in dental caries. Mediators Inflamm. 2015, 230251. doi: 10.1155/2015/230251 26538821 PMC4619960

[B49] Ferrer M. D. López-López A. Nicolescu T. Perez-Vilaplana S. Boix-Amorós A. Dzidic M.. (2020). Topic application of the probiotic *Streptococcus dentisani* improves clinical and microbiological parameters associated with oral health. Front. Cell. Infect. Microbiol. 10. doi: 10.3389/fcimb.2020.00465 PMC7488176 32984080

[B50] Galler K. M. Weber M. Korkmaz Y. Widbiller M. Feuerer M. (2021). Inflammatory response mechanisms of the dentine-pulp complex and the periapical tissues. Int. J. Mol. Sci. 22, 1480. doi: 10.3390/ijms22031480 33540711 PMC7867227

[B51] Ganguly T. Kajfasz J. K. Abranches J. Lemos J. A. (2020). Regulatory circuits controlling Spx levels in *Streptococcus mutans* . Mol. Microbiol. 114, 109–126. doi: 10.1111/mmi.14499 32189382 PMC7367440

[B52] Gomez A. Nelson K. E. (2017). The oral microbiome of children: development, disease, and implications beyond oral health. Microb. Ecol. 73, 492–503. doi: 10.1007/s00248-016-0854-1 27628595 PMC5274568

[B53] Gourieux B. Al-Okla S. Schöller-Guinard M. Klein J. Sibilia J. Wachsmann D. (2001). Pro-inflammatory cytokine production by synoviocytes following exposure to protein I/II, a modulin from oral streptococci. FEMS Immunol. Med. Microbiol. 30, 13–19. doi: 10.1111/fim.2001.30.issue-1 11172986

[B54] Graves D. T. Oates T. Garlet G. P. (2011). Review of osteoimmunology and the host response in endodontic and periodontal lesions. J. Oral Microbiol. 3, 5304. doi: 10.3402/jom.v3i0.5304 PMC3087239 21547019

[B55] Haffajee A. Socransky S. Patel M. Song X. (2008). Microbial complexes in supragingival plaque. Oral Microbiol. Immunol. 23, 196–205. doi: 10.1111/j.1399-302X.2007.00411.x 18402605

[B56] Hahn C.-L. Liewehr F. R. (2007a). Innate immune responses of the dental pulp to caries. J. Endodont. 33, 643–651. doi: 10.1016/j.joen.2007.01.001 17509400

[B57] Hahn C.-L. Liewehr F. R. (2007b). Relationships between caries bacteria, host responses, and clinical signs and symptoms of pulpitis. J. Endodont. 33, 213–219. doi: 10.1016/j.joen.2006.11.008 17320699

[B58] Hajishengallis G. (2011). Immune evasion strategies of *Porphyromonas gingivalis* . J. Oral Biosci. 53, 233–240. doi: 10.1016/S1349-0079(11)80006-X 22162663 PMC3231999

[B59] Hajishengallis G. (2014). Immunomicrobial pathogenesis of periodontitis: keystones, pathobionts, and host response. Trends Immunol. 35, 3–11. doi: 10.1016/j.it.2013.09.001 24269668 PMC3947349

[B60] Hajishengallis G. Chavakis T. Lambris J. D. (2020). Current understanding of periodontal disease pathogenesis and targets for host-modulation therapy. Periodontol. 2000 84, 14–34. doi: 10.1111/prd.12331 32844416 PMC7457922

[B61] Hajishengallis G. Darveau R. P. Curtis M. A. (2012). The keystone-pathogen hypothesis. Nat. Rev. Microbiol. 10, 717–725. doi: 10.1038/nrmicro2873 22941505 PMC3498498

[B62] Hajishengallis G. Diaz P. I. (2020). *Porphyromonas gingivalis*: Immune subversion activities and role in periodontal dysbiosis. Curr. Oral Health Rep. 7, 12–21. doi: 10.1007/s40496-020-00249-3 33344104 PMC7747940

[B63] Hajishengallis G. Lamont R. J. (2012). Beyond the red complex and into more complexity: the polymicrobial synergy and dysbiosis (PSD) model of periodontal disease etiology. Mol. Oral Microbiol. 27, 409–419. doi: 10.1111/j.2041-1014.2012.00663.x 23134607 PMC3653317

[B64] Hajishengallis G. Lamont R. J. (2014). Breaking bad: manipulation of the host response by *Porphyromonas gingivalis* . Eur. J. Immunol. 44, 328–338. doi: 10.1002/eji.201344202 24338806 PMC3925422

[B65] Hajishengallis G. Lamont R. J. (2016). Dancing with the stars: how choreographed bacterial interactions dictate nososymbiocity and give rise to keystone pathogens, accessory pathogens, and pathobionts. Trends Microbiol. 24, 477–489. doi: 10.1016/j.tim.2016.02.010 26968354 PMC4874887

[B66] Hajishengallis G. Liang S. Payne M. A. Hashim A. Jotwani R. Eskan M. A.. (2011). Low-abundance biofilm species orchestrates inflammatory periodontal disease through the commensal microbiota and complement. Cell Host Microbe 10, 497–506. doi: 10.1016/j.chom.2011.10.006 22036469 PMC3221781

[B67] Hajishengallis E. Parsaei Y. Klein M. I. Koo H. (2017). Advances in the microbial etiology and pathogenesis of early childhood caries. Mo.l Oral Microbiol. 32, 24–34. doi: 10.1111/omi.12152 PMC4929038 26714612

[B68] Hall M. W. Singh N. Ng K. F. Lam D. K. Goldberg M. B. Tenenbaum H. C.. (2017). Inter-personal diversity and temporal dynamics of dental, tongue, and salivary microbiota in the healthy oral cavity. NPJ Biofilms Microbiomes 3, 2. doi: 10.1038/s41522-016-0011-0 28649403 PMC5445578

[B69] Handley P. S. Correia F. F. Russell K. Rosan B. DiRienzo J. M. (2005). Association of a novel high molecular weight, serine-rich protein (SrpA) with fibril-mediated adhesion of the oral biofilm bacterium *Streptococcus cristatus* . Oral Microbiol. Immunol. 20, 131–140. doi: 10.1111/j.1399-302X.2004.00190.x 15836513 PMC3523328

[B70] Handley P. S. Harty D. W. Wyatt J. E. Brown C. R. Doran J. P. Gibbs A. C. (1987). A comparison of the adhesion, coaggregation and cell-surface hydrophobicity properties of fibrillar and fimbriate strains of *Streptococcus salivarius* . J. Gen. Microbiol. 133, 3207–3217. doi: 10.1099/00221287-133-11-3207 2895798

[B71] Hao T. Xie Z. Wang M. Liu L. Zhang Y. Wang W.. (2019). An anaerobic bacterium host system for heterologous expression of natural product biosynthetic gene clusters. Nat. Commun. 10, 3665. doi: 10.1038/s41467-019-11673-0 31413323 PMC6694145

[B72] Hasegawa Y. Mans J. J. Mao S. Lopez M. C. Baker H. V. Handfield M.. (2007). Gingival epithelial cell transcriptional responses to commensal and opportunistic oral microbial species. Infect. Immun. 75, 2540–2547. doi: 10.1128/IAI.01957-06 17307939 PMC1865734

[B73] Hong S. W. Baik J. E. Kang S. S. Yun C. H. Seo D. G. Han S. H. (2014a). Lipoteichoic acid of *Streptococcus mutans* interacts with Toll-like receptor 2 through the lipid moiety for induction of inflammatory mediators in murine macrophages. Mol. Immunol. 57, 284–291. doi: 10.1016/j.molimm.2013.10.004 24216318

[B74] Hong S. W. Seo D.-G. Baik J. E. Cho K. Yun C.-H. Han S. H. (2014b). Differential profiles of salivary proteins with affinity to *Streptococcus mutans* lipoteichoic acid in caries-free and caries-positive human subjects. Mol. Oral Microbiol. 29, 208–218. doi: 10.1111/omi.12057 24848678

[B75] Huse S. M. Ye Y. Zhou Y. Fodor A. A. (2012). A core human microbiome as viewed through 16S rRNA sequence clusters. PloS One 7, e34242. doi: 10.1371/journal.pone.0034242 22719824 PMC3374614

[B76] Jenkinson H. F. Demuth D. R. (1997). Structure, function and immunogenicity of streptococcal antigen I/II polypeptides. Mol. Microbiol. 23, 183–190. doi: 10.1046/j.1365-2958.1997.2021577.x 9044252

[B77] Jenkinson H. F. Terry S. D. McNab R. Tannock G. W. (1993). Inactivation of the gene encoding surface protein SspA in *Streptococcus gordonii* DL1 affects cell interactions with human salivary agglutinin and oral actinomyces. Infect. Immun. 61, 3199–3208. doi: 10.1128/iai.61.8.3199-3208.1993 8335350 PMC280988

[B78] Jespersgaard C. Hajishengallis G. Russell M. W. Michalek S. M. (2002). Identification and characterization of a nonimmunoglobulin factor in human saliva that inhibits *Streptococcus mutans* glucosyltransferase. Infect. Immun. 70, 1136–1142. doi: 10.1128/IAI.70.3.1136-1142.2002 11854193 PMC127793

[B79] Joyner P. M. Liu J. Zhang Z. Merritt J. Qi F. Cichewicz R. H. (2010). Mutanobactin A from the human oral pathogen *Streptococcus mutans* is a cross-kingdom regulator of the yeast-mycelium transition. Org. Biomol. Chem. 8, 5486–5489. doi: 10.1039/c0ob00579g 20852771 PMC2992086

[B80] Khorasani M. M. Hassanshahi G. Brodzikowska A. Khorramdelazad H. (2020). Role (s) of cytokines in pulpitis: latest evidence and therapeutic approaches. Cytokine 126, 154896. doi: 10.1016/j.cyto.2019.154896 31670007

[B81] Kim A. R. Ahn K. B. Kim H. Y. Seo H. S. Kum K. Y. Yun C. H.. (2017). *Streptococcus gordonii* lipoproteins induce IL-8 in human periodontal ligament cells. Mol. Immunol. 91, 218–224. doi: 10.1016/j.molimm.2017.09.009 28963931

[B82] Kim S. K. Im J. Ko E. B. Lee D. Seo H. S. Yun C. H.. (2023). Lipoteichoic acid of *Streptococcus gordonii* as a negative regulator of human dendritic cell activation. Front. Immunol. 14. doi: 10.3389/fimmu.2023.1056949 PMC10086370 37056772

[B83] Klein M. I. (2022). “"Oral streptococci,",” in Molecular Typing in Bacterial Infections, Volume I. Ed. de Filippis I. (Springer International Publishing, Cham), 125–137.

[B84] Ko E. B. Kim S. K. Seo H. S. Yun C. H. Han S. H. (2017). Serine-rich repeat adhesins contribute to *Streptococcus gordonii*-induced maturation of human dendritic cells. Front. Microbiol. 8. doi: 10.3389/fmicb.2017.00523 PMC5374164 28408901

[B85] Koga T. Okahashi N. Takahashi I. Kanamoto T. Asakawa H. Iwaki M. (1990). Surface hydrophobicity, adherence, and aggregation of cell surface protein antigen mutants of *Streptococcus mutans* serotype c. Infect. Immun. 58, 289–296. doi: 10.1128/iai.58.2.289-296.1990 2298480 PMC258453

[B86] Könönen E. Gursoy M. Gursoy U. K. (2019). Periodontitis: A multifaceted disease of tooth-supporting tissues. J. Clin. Med. 8, 1135. doi: 10.3390/jcm8081135 31370168 PMC6723779

[B87] Kreth J. Vu H. Zhang Y. Herzberg M. C. (2009). Characterization of hydrogen peroxide-induced DNA release by *Streptococcus sanguinis* and *Streptococcus gordonii* . J. Bacteriol. 191, 6281–6291. doi: 10.1128/JB.00906-09 19684131 PMC2753043

[B88] Kreth J. Zhang Y. Herzberg M. C. (2008). Streptococcal antagonism in oral biofilms: *Streptococcus sanguinis* and *Streptococcus gordonii* Interference with *Streptococcus mutans* . J. Bacteriol. 190, 4632–4640. doi: 10.1128/JB.00276-08 18441055 PMC2446780

[B89] Krisanaprakornkit S. Kimball J. R. Weinberg A. Darveau R. P. Bainbridge B. W. Dale B. A. (2000). Inducible expression of human beta-defensin 2 by *Fusobacterium nucleatum* in oral epithelial cells: multiple signaling pathways and role of commensal bacteria in innate immunity and the epithelial barrier. Infect. Immun. 68, 2907–2915. doi: 10.1128/IAI.68.5.2907-2915.2000 10768988 PMC97503

[B90] Kuboniwa M. Lamont R. J. (2010). Subgingival biofilm formation. Periodontol. 2000 52, 38–52. doi: 10.1111/(ISSN)1600-0757 20017794 PMC3665295

[B91] Kuboniwa M. Tribble G. D. James C. E. Kilic A. O. Tao L. Herzberg M. C.. (2006). *Streptococcus gordonii* utilizes several distinct gene functions to recruit *Porphyromonas gingivalis* into a mixed community. Mol. Microbiol. 60, 121–139. doi: 10.1111/j.1365-2958.2006.05099.x 16556225

[B92] Kuryłek A. Stasiak M. Kern-Zdanowicz I. (2022). Virulence factors of *Streptococcus anginosus* – a molecular perspective. Front. Microbiol. 13. doi: 10.3389/fmicb.2022.1025136 PMC9643698 36386673

[B93] Lamont R. J. Gil S. Demuth D. R. Malamud D. Rosan B. (1994). Molecules of *Streptococcus gordonii* that bind to *Porphyromonas gingivalis* . Microbiology 140, 867–872. doi: 10.1099/00221287-140-4-867 8012603

[B94] Lamont R. J. Koo H. Hajishengallis G. (2018). The oral microbiota: dynamic communities and host interactions. Nat. Rev. Microbiol. 16, 745–759. doi: 10.1038/s41579-018-0089-x 30301974 PMC6278837

[B95] Lang M. L. Zhu L. Kreth J. (2010). Keeping the bad bacteria in check: interactions of the host immune system with oral cavity biofilms. Endodont. Topics 22, 17–32. doi: 10.1111/j.1601-1546.2012.00278.x

[B96] Lemos J. A. Palmer S. R. Zeng L. Wen Z. T. Kajfasz J. K. Freires I. A.. (2019). The biology of *Streptococcus mutans* . Microbiol. Spectr. 7, GPP3-0051-2018. doi: 10.1128/microbiolspec.GPP3-0051-2018 PMC6615571 30657107

[B97] Li Z. R. Sun J. Du Y. Pan A. Zeng L. Maboudian R.. (2021). Mutanofactin promotes adhesion and biofilm formation of cariogenic *Streptococcus mutans* . Nat. Chem. Biol. 17 (5), 576–584. doi: 10.1038/s41589-021-00745-2 33664521

[B98] Lima A. R. Ganguly T. Walker A. R. Acosta N. Francisco P. A. Pileggi R.. (2020). Phenotypic and genotypic characterization of *Streptococcus mutans* strains isolated from endodontic infections. J. Endodont. 46, 1876–1883. doi: 10.1016/j.joen.2020.09.002 PMC7686129 32919986

[B99] Lima B. P. Kho K. Nairn B. L. Davies J. R. Svensäter G. Chen R.. (2019). *Streptococcus gordonii* type I lipoteichoic acid contributes to surface protein biogenesis. mSphere 4, e00814-19. doi: 10.1128/mSphere.00814-19 31801844 PMC6893214

[B100] Liu L. Hao T. Xie Z. Horsman G. P. Chen Y. (2016). Genome mining unveils widespread natural product biosynthetic capacity in human oral microbe *Streptococcus mutans* . Sci. Rep. 6, 37479. doi: 10.1038/srep37479 27869143 PMC5116633

[B101] Liu J. Sun L. Liu W. Guo L. Liu Z. Wei X.. (2017). A nuclease from *Streptococcus mutans* facilitates biofilm dispersal and escape from killing by neutrophil extracellular traps. Front. Cell. Infect. Microbiol. 7. doi: 10.3389/fcimb.2017.00097 PMC5368189 28401067

[B102] Liu T. Yang R. Zhou J. Lu X. Yuan Z. Wei X.. (2021). Interactions between *Streptococcus gordonii* and *Fusobacterium nucleatum* altered bacterial transcriptional profiling and attenuated the immune responses of macrophages. Front. Cell. Infect. Microbiol. 11. doi: 10.3389/fcimb.2021.783323 PMC8776643 35071038

[B103] Loos B. G. Van Dyke T. E. (2020). The role of inflammation and genetics in periodontal disease. Periodontol. 2000 83, 26–39. doi: 10.1111/prd.12297 32385877 PMC7319430

[B104] Love R. M. McMillan M. D. Jenkinson H. F. (1997). Invasion of dentinal tubules by oral streptococci is associated with collagen recognition mediated by the antigen I/II family of polypeptides. Infect. Immun. 65, 5157–5164. doi: 10.1128/iai.65.12.5157-5164.1997 9393810 PMC175743

[B105] MacDonald K. W. Chanyi R. M. Macklaim J. M. Cadieux P. A. Reid G. Burton J. P. (2021). *Streptococcus salivarius* inhibits immune activation by periodontal disease pathogens. BMC Oral Health 21, 245. doi: 10.1186/s12903-021-01606-z 33962608 PMC8103598

[B106] Maeda K. Tribble G. D. Tucker C. M. Anaya C. Shizukuishi S. Lewis J. P.. (2008). A *Porphyromonas gingivalis* tyrosine phosphatase is a multifunctional regulator of virulence attributes. Mol. Microbiol. 69, 1153–1164. doi: 10.1111/j.1365-2958.2008.06338.x 18573179 PMC2537464

[B107] Mager D. L. Ximenez-Fyvie L. A. Haffajee A. D. Socransky S. S. (2003). Distribution of selected bacterial species on intraoral surfaces. J. Clin. Periodontol. 30, 644–654. doi: 10.1034/j.1600-051X.2003.00376.x 12834503

[B108] Manzer H. S. Nobbs A. H. Doran K. S. (2020). The multifaceted nature of streptococcal antigen I/II proteins in colonization and disease pathogenesis. Front. Microbiol. 11. doi: 10.3389/fmicb.2020.602305 PMC7732690 33329493

[B109] Mark Welch J. L. Ramírez-Puebla S. T. Borisy G. G. (2020). Oral microbiome geography: micron-scale habitat and niche. Cell Host Microbe 28, 160–168. doi: 10.1016/j.chom.2020.07.009 32791109 PMC7604680

[B110] Marsh P. D. Zaura E. (2017). Dental biofilm: ecological interactions in health and disease. J. Clin. Periodontol. 44, S12–S22. doi: 10.1111/jcpe.12679 28266111

[B111] Martin V. Kleschyov A. L. Klein J. P. Beretz A. (1997). Induction of nitric oxide production by polyosides from the cell walls of *Streptococcus mutans* OMZ 175, a gram-positive bacterium, in the rat aorta. Infect. Immun. 65, 2074–2079. doi: 10.1128/iai.65.6.2074-2079.1997 9169734 PMC175286

[B112] Martorano-Fernandes L. Brito A. C. M. Araújo E. Almeida L. Wei X.-Q. Williams D. W.. (2023). Epithelial responses and *Candida albicans* pathogenicity are enhanced in the presence of oral streptococci. Braz. Dent. J. 34, 73–81. doi: 10.1590/0103-6440202305420 37466528 PMC10355268

[B113] Matsushima H. Kumagai Y. Vandenbon A. Kataoka H. Kadena M. Fukamachi H.. (2017). Microarray analysis of macrophage response to infection with *Streptococcus oralis* reveals the immunosuppressive effect of hydrogen peroxide. Biochem. Biophys. Res. Commun. 485, 461–467. doi: 10.1016/j.bbrc.2017.02.048 28202416

[B114] McNab R. Holmes A. R. Clarke J. M. Tannock G. W. Jenkinson H. F. (1996). Cell surface polypeptide CshA mediates binding of *Streptococcus gordonii* to other oral bacteria and to immobilized fibronectin. Infect. Immun. 64, 4204–4210. doi: 10.1128/iai.64.10.4204-4210.1996 8926089 PMC174357

[B115] Medapati M. R. Singh N. Bhagirath A. Y. Duan K. Triggs-Raine B. Batista E. L. Jr.. (2021). Bitter taste receptor T2R14 detects quorum sensing molecules from cariogenic Streptococcus mutans and mediates innate immune responses in gingival epithelial cells. FASEB J. 35, e21375. doi: 10.1096/fj.202000208R 33559200

[B116] Miller J. H. Avilés-Reyes A. Scott-Anne K. Gregoire S. Watson G. E. Sampson E.. (2015). The collagen binding protein Cnm contributes to oral colonization and cariogenicity of *Streptococcus mutans* OMZ175. Infect. Immun. 83, 2001–2010. doi: 10.1128/IAI.03022-14 25733523 PMC4399078

[B117] Mira A. (2018). Oral microbiome studies: potential diagnostic and therapeutic implications. Adv. Dent. Res. 29, 71–77. doi: 10.1177/0022034517737024 29355422

[B118] Mitchell J. (2011). *Streptococcus mitis*: walking the line between commensalism and pathogenesis. Mol. Oral Microbiol. 26, 89–98. doi: 10.1111/j.2041-1014.2010.00601.x 21375700

[B119] Morita C. Sumioka R. Nakata M. Okahashi N. Wada S. Yamashiro T.. (2014). Cell wall-anchored nuclease of *Streptococcus sanguinis* contributes to escape from neutrophil extracellular trap-mediated bacteriocidal activity. PloS One 9, e103125. doi: 10.1371/journal.pone.0103125 25084357 PMC4118848

[B120] Mutha N. V. R. Mohammed W. K. Krasnogor N. Tan G. Y. A. Choo S. W. Jakubovics N. S. (2018). Transcriptional responses of *Streptococcus gordonii* and *Fusobacterium nucleatum* to coaggregation. Mol. Oral Microbiol. 33, 450–464. doi: 10.1111/omi.12248 30329223

[B121] Myers S. Do T. Meade J. L. Tugnait A. Vernon J. J. Pistolic J.. (2021). Immunomodulatory streptococci that inhibit CXCL8 secretion and NFkappaB activation are common members of the oral microbiota. J. Med. Microbiol. 70, 1329. doi: 10.1099/jmm.0.001329 PMC8346732 33734952

[B122] Nagata E. Okayama H. Ito H.-O. Yamashita Y. Inoue M. Oho T. (2006). Serotype-specific polysaccharide of *Streptococcus mutans* contributes to infectivity in endocarditis. Oral Microbiol. Immunol. 21, 420–423. doi: 10.1111/j.1399-302X.2006.00317.x 17064403

[B123] Naka S. Hatakeyama R. Takashima Y. Matsumoto-Nakano M. Nomura R. Nakano K. (2016). Contributions of *Streptococcus mutans* Cnm and PA antigens to aggravation of non-alcoholic steatohepatitis in mice. Sci. Rep. 6, 36886. doi: 10.1038/srep36886 27833139 PMC5105074

[B124] Naka S. Matsuoka D. Goto K. Misaki T. Nagasawa Y. Ito S.. (2022). Cnm of *Streptococcus mutans* is important for cell surface structure and membrane permeability. Front. Cell. Infect. Microbiol. 12. doi: 10.3389/fcimb.2022.994014 PMC9513430 36176579

[B125] Naka S. Nomura R. Takashima Y. Okawa R. Ooshima T. Nakano K. (2014). A specific *Streptococcus mutans* strain aggravates non-alcoholic fatty liver disease. Oral Dis. 20, 700–706. doi: 10.1111/odi.12191 25360469

[B126] Naka S. Wato K. Hatakeyama R. Okawa R. Nomura R. Nakano K. (2018). Longitudinal comparison of *Streptococcus mutans*-induced aggravation of non-alcoholic steatohepatitis in mice. J. Oral Microbiol. 10, 1428005. doi: 10.1080/20002297.2018.1428005 29503703 PMC5795759

[B127] Nakano K. Hokamura K. Taniguchi N. Wada K. Kudo C. Nomura R.. (2011). The collagen-binding protein of *Streptococcus mutans* is involved in haemorrhagic stroke. Nat. Commun. 2, 485. doi: 10.1038/ncomms1491 21952219 PMC3220351

[B128] Nakano K. Nomura R. Taniguchi N. Lapirattanakul J. Kojima A. Naka S.. (2010). Molecular characterization of *Streptococcus mutans* strains containing the cnm gene encoding a collagen-binding adhesin. Arch. Oral Biol. 55, 34–39. doi: 10.1016/j.archoralbio.2009.11.008 20005510

[B129] Nakano K. Ooshima T. (2009). Serotype classification of *Streptococcus mutans* and its detection outside the oral cavity. Future Microbiol. 4, 891–902. doi: 10.2217/fmb.09.64 19722842

[B130] Narayanan L. L. Vaishnavi C. (2010). Endodontic microbiology. J. Conserv. Dent. 13, 233–239. doi: 10.4103/0972-0707.73386 21217951 PMC3010028

[B131] Negrini T. C. Duque C. Vizoto N. L. Stipp R. N. Mariano F. S. Höfling J. F.. (2012). Influence of VicRK and CovR on the interactions of *Streptococcus mutans* with phagocytes. Oral Dis. 18, 485–493. doi: 10.1111/j.1601-0825.2011.01896.x 22233463

[B132] Nobbs A. H. Jenkinson H. F. Everett D. B. (2015). Generic determinants of *Streptococcus* colonization and infection. Infect. Gen. Evol. 33, 361–370. doi: 10.1016/j.meegid.2014.09.018 25246075

[B133] Nobbs A. H. Lamont R. J. Jenkinson H. F. (2009). *Streptococcus* adherence and colonization. Microbiol. Mol. Biol. Rev. 73, 407–450. doi: 10.1128/MMBR.00014-09 19721085 PMC2738137

[B134] Nomura R. Naka S. Nemoto H. Inagaki S. Taniguchi K. Ooshima T.. (2013). Potential involvement of collagen-binding proteins of *Streptococcus mutans* in infective endocarditis. Oral Dis. 19, 387–393. doi: 10.1111/odi.12016 22998492

[B135] Okahashi N. Nakata M. Kuwata H. Kawabata S. (2016). *Streptococcus oralis* induces lysosomal impairment of macrophages via bacterial hydrogen peroxide. Infect. Immun. 84, 2042–2050. doi: 10.1128/IAI.00134-16 27113357 PMC4936348

[B136] Okahashi N. Nakata M. Kuwata H. Kawabata S. (2022). Oral *mitis* group streptococci: A silent majority in our oral cavity. Microbiol. Immunol. 66, 539–551. doi: 10.1111/1348-0421.13028 36114681

[B137] Okahashi N. Nakata M. Sumitomo T. Terao Y. Kawabata S. (2013). Hydrogen peroxide produced by oral streptococci induces macrophage cell death. PloS One 8, e62563. doi: 10.1371/journal.pone.0062563 23658745 PMC3643943

[B138] Okahashi N. Okinaga T. Sakurai A. Terao Y. Nakata M. Nakashima K.. (2011). *Streptococcus sanguinis* induces foam cell formation and cell death of macrophages in association with production of reactive oxygen species. FEMS Microbiol. Lett. 323, 164–170. doi: 10.1111/fml.2011.323.issue-2 22092716

[B139] Okahashi N. Sumitomo T. Nakata M. Sakurai A. Kuwata H. Kawabata S. (2014). Hydrogen peroxide contributes to the epithelial cell death induced by the oral mitis group of streptococci. PloS One 9, e88136. doi: 10.1371/journal.pone.0088136 24498253 PMC3909332

[B140] Parducho K. R. Beadell B. Ybarra T. K. Bush M. Escalera E. Trejos A. T.. (2020). The antimicrobial peptide human beta-defensin 2 inhibits biofilm production of *Pseudomonas aeruginosa* without compromising metabolic activity. Front. Immunol. 11. doi: 10.3389/fimmu.2020.00805 PMC7225314 32457749

[B141] Park O. J. Kim A. R. So Y. J. Im J. Ji H. J. Ahn K. B.. (2021). Induction of apoptotic cell death by oral streptococci in human periodontal ligament cells. Front. Microbiol. 12. doi: 10.3389/fmicb.2021.738047 PMC8551966 34721337

[B142] Park O. J. Kwon Y. Park C. So Y. J. Park T. H. Jeong S.. (2020). *Streptococcus gordonii*: Pathogenesis and host response to its cell wall components. Microorganisms 8, 1852. doi: 10.3390/microorganisms8121852 PMC7761167 33255499

[B143] Park O.-J. Yi H. Jeon J. Kang S.-S. Koo K.-T. Kum K.-Y.. (2015). Pyrosequencing analysis of subgingival microbiota in distinct periodontal conditions. J. Dent. Res. 94, 921–927. doi: 10.1177/0022034515583531 25904141

[B144] Patel M. (2020). Dental caries vaccine: are we there yet? Lett. Appl. Microbiol. 70, 2–12. doi: 10.1111/lam.13218 31518435

[B145] Percy M. G. Gründling A. (2014). Lipoteichoic acid synthesis and function in Gram-positive bacteria. Annu. Rev. Microbiol. 68, 81–100. doi: 10.1146/annurev-micro-091213-112949 24819367

[B146] Pilarczyk-Zurek M. Sitkiewicz I. Koziel J. (2022). The clinical view on *Streptococcus anginosus* group – Opportunistic pathogens coming out of hiding. Front. Microbiol. 13. doi: 10.3389/fmicb.2022.956677 PMC9309248 35898914

[B147] Plummer C. Wu H. Kerrigan S. W. Meade G. Cox D. Ian Douglas C. W. (2005). A serine-rich glycoprotein of *Streptococcus sanguis* mediates adhesion to platelets via GPIb. Br. J. Haematol. 129, 101–109. doi: 10.1111/j.1365-2141.2005.05421.x 15801962

[B148] Pultar F. Hansen M. E. Wolfrum S. Böselt L. Fróis-Martins R. Bloch S.. (2021). Mutanobactin D from the human microbiome: Total synthesis, configurational assignment, and biological evaluation. J. Am. Chem. Soc 143, 10389–10402. doi: 10.1021/jacs.1c04825 34212720

[B149] Ramsey M. M. Rumbaugh K. P. Whiteley M. (2011). Metabolite cross-feeding enhances virulence in a model polymicrobial infection. PloS Pathog. 7, e1002012. doi: 10.1371/journal.ppat.1002012 21483753 PMC3069116

[B150] Ray C. A. Gfell L. E. Buller T. L. Gregory R. L. (1999). Interactions of *Streptococcus mutans* fimbria-associated surface proteins with salivary components. Clin. Diagn. Lab. Immunol. 6, 400–404. doi: 10.1128/CDLI.6.3.400-404.1999 10225843 PMC103730

[B151] Richards V. P. Alvarez A. J. Luce A. R. Bedenbaugh M. Mitchell M. L. Burne R. A.. (2017). Microbiomes of site-specific dental plaques from children with different caries status. Infect. Immun. 85, e00106–e00117. doi: 10.1128/IAI.00106-17 28507066 PMC5520424

[B152] Rijn I. V. D. Bleiweis A. S. (1973). Antigens of *Streptococcus mutans* I. Characterization of a serotype-specific determinant from *Streptococcus mutans* . Infect. Immun. 7, 795–804. doi: 10.1128/iai.7.5.795-804.1973 4128669 PMC422763

[B153] Rølla G. Oppermann R. V. Bowen W. H. Ciardi J. E. Knox K. W. (2009). High amounts of lipoteichoic acid in sucrose-induced plaque in *vivo* . Caries Res. 14, 235–238. doi: 10.1159/000260459 6769589

[B154] Ruhl S. Cisar J. O. Sandberg A. L. (2000). Identification of polymorphonuclear leukocyte and HL-60 cell receptors for adhesins of *Streptococcus gordonii* and *Actinomyces naeslundii* . Infect. Immun. 68, 6346–6354. doi: 10.1128/IAI.68.11.6346-6354.2000 11035744 PMC97718

[B155] Sampaio-Maia B. Monteiro-Silva F. (2014). Acquisition and maturation of oral microbiome throughout childhood: An update. Dent. Res. J. 11, 291–301. PMC4119360 25097637

[B156] Scott D. A. Krauss J. (2012). Neutrophils in periodontal inflammation. Front. Oral Biol. 15, 56–83. doi: 10.1159/000329672 22142957 PMC3335266

[B157] Sherman J. M. (1937). The streptococci. Bacteriol. Rev. 1, 3–97. doi: 10.1128/br.1.1.3-97.1937 16350049 PMC440821

[B158] Shun C. T. Lu S. Y. Yeh C. Y. Chiang C. P. Chia J. S. Chen J. Y. (2005). Glucosyltransferases of viridans streptococci are modulins of interleukin-6 induction in infective endocarditis. Infect. Immun. 73, 3261–3270. doi: 10.1128/IAI.73.6.3261-3270.2005 15908350 PMC1111834

[B159] Silhavy T. J. Kahne D. Walker S. (2010). The bacterial cell envelope. Cold Spring Harbor Perspect. Biol. 2, a000414–a000414. doi: 10.1101/cshperspect.a000414 PMC2857177 20452953

[B160] Silverman R. J. Nobbs A. H. Vickerman M. M. Barbour M. E. Jenkinson H. F. (2010). Interaction of *Candida albicans* cell wall Als3 protein with *Streptococcus gordonii* SspB adhesin promotes development of mixed-species communities. Infect. Immun. 78, 4644–4652. doi: 10.1128/IAI.00685-10 20805332 PMC2976310

[B161] Simon-Soro A. Ren Z. Krom B. P. Hoogenkamp M. A. Cabello-Yeves P. J. Daniel S. G.. (2022). Polymicrobial aggregates in human saliva build the oral biofilm. mBio 13, e0013122. doi: 10.1128/mbio.00131-22 35189700 PMC8903893

[B162] Singh A. K. Woodiga S. A. Grau M. A. King S. J. (2017). *Streptococcus oralis* neuraminidase modulates aherence to multiple crbohydrates on platelets. Infect. Immun. 85, e00774–e00716. doi: 10.1128/IAI.00774-16 27993975 PMC5328485

[B163] Sitkiewicz I. (2018). How to become a killer, or is it all accidental? Virulence strategies in oral streptococci. Mol. Oral Microbiol. 33, 1–12. doi: 10.1111/omi.12192 28727895

[B164] Sliepen I. Van Damme J. Van Essche M. Loozen G. Quirynen M. Teughels W. (2009a). Microbial interactions influence inflammatory host cell responses. J. Dent. Res. 88, 1026–1030. doi: 10.1177/0022034509347296 19828891

[B165] Sliepen I. Van Essche M. Loozen G. Van Eldere J. Quirynen M. Teughels W. (2009b). Interference with *Aggregatibacter actinomycetemcomitans*: colonization of epithelial cells under hydrodynamic conditions. Oral Microbiol. Immunol. 24, 390–395. doi: 10.1111/j.1399-302X.2009.00531.x 19702952

[B166] Soell M. Holveck F. Schöller M. Wachsmann R. D. Klein J. P. (1994). Binding of *Streptococcus mutans* SR protein to human monocytes: production of tumor necrosis factor, interleukin 1, and interleukin 6. Infect. Immun. 62, 1805–1812. doi: 10.1128/iai.62.5.1805-1812.1994 8168943 PMC186412

[B167] Soell M. Lett E. Holveck F. Schöller M. Wachsmann D. Klein J. P. (1995). Activation of human monocytes by streptococcal rhamnose glucose polymers is mediated by CD14 antigen, and mannan binding protein inhibits TNF-alpha release. J. Immunol. 154, 851–860. doi: 10.4049/jimmunol.154.2.851 7529289

[B168] Stinson M. W. Nisengard R. J. Bergey E. J. (1980). Binding of streptococcal antigens to muscle tissue in *vitro* . Infect. Immun. 27, 604–613. doi: 10.1128/iai.27.2.604-613.1980 6991420 PMC550807

[B169] Sugawara S. Arakaki R. Rikiishi H. Takada H. (1999). Lipoteichoic acid acts as an antagonist and an agonist of lipopolysaccharide on human gingival fibroblasts and monocytes in a CD14-dependent manner. Infect. Immun. 67, 1623–1632. doi: 10.1128/IAI.67.4.1623-1632.1999 10084995 PMC96505

[B170] Sugiyama A. Arakaki R. Ohnishi T. Arakaki N. Daikuhara Y. Takada H. (1996). Lipoteichoic acid and interleukin 1 stimulate synergistically production of hepatocyte growth factor (scatter factor) in human gingival fibroblasts in culture. Infect. Immun. 64, 1426–1431. doi: 10.1128/iai.64.4.1426-1431.1996 8606111 PMC173936

[B171] Sumioka R. Nakata M. Okahashi N. Li Y. Wada S. Yamaguchi M.. (2017). *Streptococcus sanguinis* induces neutrophil cell death by production of hydrogen peroxide. PloS One 12, e0172223. doi: 10.1371/journal.pone.0172223 28222125 PMC5319702

[B172] Takahashi Y. Konishi K. Cisar J. O. Yoshikawa M. (2002). Identification and characterization of hsa, the gene encoding the sialic acid-binding adhesin of *Streptococcus gordonii* DL1. Infect. Immun. 70, 1209–1218. doi: 10.1128/IAI.70.3.1209-1218.2002 11854202 PMC127787

[B173] Takamatsu D. Bensing B. A. Cheng H. Jarvis G. A. Siboo I. R. López J. A.. (2005). Binding of the *Streptococcus gordonii* surface glycoproteins GspB and Hsa to specific carbohydrate structures on platelet membrane glycoprotein Ibalpha. Mol. Microbiol. 58, 380–392. doi: 10.1111/j.1365-2958.2005.04830.x 16194227

[B174] Tang X. Kudo Y. Baker J. L. LaBonte S. Jordan P. A. McKinnie S. M. K.. (2020). Cariogenic *Streptococcus mutans* produces tetramic acid strain-specific antibiotics that impair commensal colonization. ACS Infect. Dis. 6, 563–571. doi: 10.1021/acsinfecdis.9b00365 31906623 PMC7150634

[B175] Tang Y. L. Sim T. S. Tan K. S. (2022). Oral streptococci subvert the host innate immune response through hydrogen peroxide. Sci. Rep. 12, 656. doi: 10.1038/s41598-021-04562-4 35027607 PMC8758666

[B176] Teughels W. Kinder Haake S. Sliepen I. Pauwels M. Van Eldere J. Cassiman J. J.. (2007). Bacteria interfere with *A. actinomycetemcomitans* colonization. J. Dent. Res. 86, 611–617. doi: 10.1177/154405910708600706 17586706

[B177] Thurnheer T. Belibasakis G. N. Bostanci N. (2014). Colonisation of gingival epithelia by subgingival biofilms in *vitro*: Role of "red complex" bacteria. Arch. Oral Biol. 59, 977–986. doi: 10.1016/j.archoralbio.2014.05.023 24949828

[B178] Tsuda H. Yamashita Y. Toyoshima K. Yamaguchi N. Oho T. Nakano Y.. (2000). Role of serotype-specific polysaccharide in the resistance of *Streptococcus mutans* to phagocytosis by human polymorphonuclear leukocytes. Infect. Immun. 68, 644–650. doi: 10.1128/IAI.68.2.644-650.2000 10639428 PMC97187

[B179] Urano-Tashiro Y. Takahashi Y. Oguchi R. Konishi K. (2016). Two arginine residues of *Streptococcus gordonii* sialic acid-binding adhesin hsa are essential for interaction to host cell receptors. PloS One 11, e0154098. doi: 10.1371/journal.pone.0154098 27101147 PMC4839618

[B180] Vacca-Smith A. M. Jones C. A. Levine M. J. Stinson M. W. (1994). Glucosyltransferase mediates adhesion of *Streptococcus gordonii* to human endothelial cells in *vitro* . Infect. Immun. 62, 2187–2194. doi: 10.1128/iai.62.6.2187-2194.1994 8188339 PMC186496

[B181] Van Hoogmoed C. G. Geertsema-Doornbusch G. I. Teughels W. Quirynen M. Busscher H. J. van der Mei H. C. (2008). Reduction of periodontal pathogens adhesion by antagonistic strains. Oral Microbiol. Immunol. 23, 43–48. doi: 10.1111/j.1399-302X.2007.00388.x 18173797

[B182] Vernier A. Diab M. Soell M. Haan-Archipoff G. Beretz A. Wachsmann D.. (1996). Cytokine production by human epithelial and endothelial cells following exposure to oral viridans streptococci involves lectin interactions between bacteria and cell surface receptors. Infect. Immun. 64, 3016–3022. doi: 10.1128/iai.64.8.3016-3022.1996 8757828 PMC174182

[B183] Vernier-Georgenthum A. al-Okla S. Gourieux B. Klein J. P. Wachsmann D. (1998). Protein I/II of oral viridans streptococci increases expression of adhesion molecules on endothelial cells and promotes transendothelial migration of neutrophils in *vitro* . Cell. Immunol. 187, 145–150. doi: 10.1006/cimm.1998.1327 9732703

[B184] Wang X. Du L. You J. King J. B. Cichewicz R. H. (2012). Fungal biofilm inhibitors from a human oral microbiome-derived bacterium. Org. Biomol. Chem. 10, 2044–2050. doi: 10.1039/c2ob06856g 22281750

[B185] Wang P. L. Shirasu S. Daito M. Ohura K. (2001). *Streptococcus mutans* lipoteichoic acid-induced apoptosis in cultured dental pulp cells from human deciduous teeth. Biochem. Biophys. Res. Commun. 281, 957–961. doi: 10.1006/bbrc.2001.4451 11237754

[B186] Weerkamp A. H. van der Mei H. C. Slot J. (1987). Relationship of cell surface morphology and composition of *Streptococcus salivarius* K^+^ to adherence and hydrophobicity. Infect. Immun. 55, 438–445. doi: 10.1128/iai.55.2.438-445.1987 3804445 PMC260347

[B187] Whitmore S. E. Lamont R. J. (2011). The pathogenic persona of community-associated oral streptococci. Mol. Microbiol. 81, 305–314. doi: 10.1111/j.1365-2958.2011.07707.x 21635580 PMC3248243

[B188] Wu H. Bu S. Newell P. Chen Q. Fives-Taylor P. (2007). Two gene determinants are differentially involved in the biogenesis of Fap1 precursors in *Streptococcus parasanguis* . J. Bacteriol. 189, 1390–1398. doi: 10.1128/JB.00836-06 16997950 PMC1797361

[B189] Wu C. Cichewicz R. Li Y. Liu J. Roe B. Ferretti J.. (2010). Genomic island TnSmu2 of *Streptococcus mutans* harbors a nonribosomal peptide synthetase-polyketide synthase gene cluster responsible for the biosynthesis of pigments involved in oxygen and H_2_O_2_ tolerance. Appl. Environ. Microbiol. 76, 5815–5826. doi: 10.1128/AEM.03079-09 20639370 PMC2935078

[B190] Xu H. Sobue T. Bertolini M. Thompson A. Dongari-Bagtzoglou A. (2016). *Streptococcus oralis* and *Candida albicans* synergistically activate μ-Calpain to degrade E-cadherin from oral epithelial junctions. J. Infect. Dis. 214, 925–934. doi: 10.1093/infdis/jiw201 27190184 PMC4996146

[B191] Xu H. Sobue T. Thompson A. Xie Z. Poon K. Ricker A.. (2014). Streptococcal co-infection augments *Candida* pathogenicity by amplifying the mucosal inflammatory response. Cell. Microbiol. 16, 214–231. doi: 10.1111/cmi.12216 24079976 PMC3956708

[B192] Yang C. Scoffield J. Wu R. Deivanayagam C. Zou J. Wu H. (2018). Antigen I/II mediates interactions between *Streptococcus mutans* and *Candida albicans* . Mol. Oral Microbiol. 33, 283–291. doi: 10.1111/omi.12223 29570954 PMC6041162

[B193] Yumoto H. Hirota K. Hirao K. Ninomiya M. Murakami K. Fujii H.. (2019). The pathogenic factors from oral streptococci for systemic diseases. Int. J. Mol. Sci. 20, 4571. doi: 10.3390/ijms20184571 31540175 PMC6770522

[B194] Zhang G. Chen R. Rudney J. D. (2008). *Streptococcus cristatus* attenuates *Fusobacterium nucleatum*-induced interleukin-8 expression in oral epithelial cells. J. Periodont. Res. 43, 408–416. doi: 10.1111/j.1600-0765.2007.01057.x 18942189

[B195] Zhang G. Chen R. Rudney J. D. (2011). *Streptococcus cristatus* modulates the *Fusobacterium nucleatum*-induced epithelial interleukin-8 response through the nuclear factor-kappa B pathway. J. Periodont. Res. 46, 558–567. doi: 10.1111/j.1600-0765.2011.01373.x 21521225

[B196] Zhu L. Kreth J. (2012). The role of hydrogen peroxide in environmental adaptation of oral microbial communities. Oxid. Med. Cell. Longev. 2012, 717843. doi: 10.1155/2012/717843 22848782 PMC3405655

[B197] Zvanych R. Lukenda N. Li X. Kim J. J. Tharmarajah S. Magarvey N. A. (2015). Systems biosynthesis of secondary metabolic pathways within the oral human microbiome member *Streptococcus mutans* . Mol. Biosyst. 11, 97–104. doi: 10.1039/C4MB00406J 25209237

